# Supplementary material for: Same streams in a different forest? Investigations of forest harvest legacies and future trajectories across 30 years of stream habitat monitoring on the Tongass National Forest, Alaska
Source: PLoS One. 2024 Jul 10;19(7):e0301723. doi: 10.1371/journal.pone.0301723 (PMC11236125; doi:10.1371/journal.pone.0301723)
Supplement: S1 File — S1 Table. Stream habitat metrics that were used as response variables in multivariate and univariate statistical models and tests. Metrics are grouped into different categories (bold italic headings). Metric names are those used in the manuscript text; abbreviations in parentheses are used in tables and figures. S2 Table. Minimum size thresholds for wood pieces to be considered “key wood”. CBW- Channel Bed Width.S3 Table. Best-supported models for each variable. Refer to Tables 1 and 2 for variable abbreviations. “TF” specifies whether response variable was natural log transformed (LN) or not (“No”). N is the sample size of available surveys used in that model. MR2 is the marginal pseudo R2 representing the amount of variance explained by fixed variables and CR2 and the conditional pseudo R2 representing the amount of variance explained by the fixed and conditional variables. See S1 Table and Table 1 for variable abbreviation explanations. S4 Table. Descriptions of channel process group classes. S5 Table. Multi Response Permutation Procedure (MRPP) pairwise comparisons for three management classes and two process groups. A is the effect size and P is the p- value for the test. The significance threshold was adjusted for multiple comparisons using Bonferroni correction 0.05/4 tests = 0.013. (DOCX) [file pone.0301723.s001.docx]

**Same streams in a diff­­erent forest? Investigations of forest harvest legacies and future trajectories across 30 years of stream habitat monitoring on the Tongass National Forest, Alaska**

Moore, Michael. J. ^1^, Flitcroft, Rebecca. L.^2*^, Tucker, Emil.^3^, Prussian, Katherine. M. ^4^, Claeson, Shannon. M.^5^

Short Title: Impacts of Forest Harvest Legacies on Stream Habitat in the Tongass National Forest, Alaska

^1^ U.S. Geological Survey, Iowa Cooperative Fish and Wildlife Research Unit, 339 Science II, 2310 Pammel Drive, Ames, IA, United States of America

^2^ U.S. Forest Service, Pacific Northwest Research Station, Corvallis Forestry Sciences Laboratory, 3200 SW Jefferson Way, Corvallis, OR, United States of America

^3^ U.S. Forest Service, Tongass National Forest, 123 Scow Bay Loop Road, Petersburg, AK, United States of America

^4^ U.S. Forest Service, Tongass National Forest, 2108 Halibut Point Road, Sitka, AK., United States of America

^5^ U.S. Forest Service, Pacific Northwest Research Station, Wenatchee Forestry Sciences Laboratory 1133 N Western Ave, Wenatchee, WA, United States of America

*Corresponding Author

E-mail: rebecca.flitcroft@usda.gov (RF)

Any use of trade, firm, or product names is for descriptive purposes only and does not imply endorsement by the U.S. Government.

**Supporting information**

**Table S1.** Stream habitat metrics that were used as response variables in multivariate and univariate statistical models and tests. Metrics are grouped into different categories (bold italic headings). Metric names are those used in the manuscript text; abbreviations in parentheses are used in tables and figures.

| **Stream habitat metric**  **(abbreviation)** | **Description** | **Relevance to biota/ habitat condition** |
| --- | --- | --- |
| ***Channel morphology*** |  |  |
| Hydraulic radius (radius) | Channel cross section area / cross section perimeter | General descriptor of channel cross-section shape. For a given cross-sectional area, relatively flat, shallow channels have a low radius, and deep, narrow channels have a high radius. |
| Width:depth (WD) | Mean bankfull width / mean bankfull depth | Measure of channel disturbance. Where there is channel widening or shallowing following floods/debris flows/loss of riparian vegetation, values will be high relative to reference conditions, but values will be low where channel entrenchment is occurring. Better condition produces intermediate values. Shallower depth can increase stream temperature. |
| Undercut bank density (UB) | Length of undercut banks / length of surveyed reach (m). Where vertical water depth is >0.3 m, measured as contiguous lengths separately for each bank and combined. | Undercut banks, where wetted at low to medium flow, are important cover habitat for fish. At high flows they provide refugia from center channel velocities. They are thought to be a sign of well-vegetated and intact banks. |
| ***Riffles*** |  |  |
| Riffle area | Total area in riffle habitat | Riffles provide habitat for macroinvertebrate prey production and spawning habitat for salmonids. |
| ***Pools*** |  |  |
| Pool density  (pools/km) | Total number of pools / meters surveyed * 1000 | Pools provide important rearing habitat for juvenile fishes, especially coho salmon (*Oncorhynchus kisutch*) which spend longer in freshwater as juveniles. Higher values are desirable because pools are usually more limited than salmon spawning habitat (i.e., riffles) in these streams. |
| Pool spacing (PoolSpace) | Average distance between pools expressed in units of channel width: (reach length / average width of channel bed from bottom of bank to bottom of bank) / number of pools [16]. | See above. Lower values indicate less space between pools and thus higher pool density. |
| Relative pool area (RPA) | Surficial pool area / total stream area surveyed | Larger pool area = higher RPA. Pool area is important overwintering habitat for juvenile salmonids. |
| Residual pool depth (RPD) | Maximum pool depth minus pool tail (riffle crest) depth | Deeper pools may be more complex, have lower water velocities, and be less likely to freeze over winter, making them important fish habitats. |
| Residual pool depth/ channel bed width (RPD/CBW) | Residual pool depth (as defined above) normalized by bed width | See above; adjusts RPD for stream size. |
| Pool size (PoolSize) | Mean RPD / mean bankfull depth [31] | A measure of pool quality to describe the depth of pools relative to other stream features. Higher values are better because deep pool habitat is usually limited by lack of large wood at disturbed sites. |
| ***Wood*** |  |  |
| Large wood density (LW/m) | Number of large wood pieces (>1 m in length and 0.1 m in diameter) within the bankfull channel / length of survey reach (m) | Large wood provides macroinvertebrate habitat, juvenile fish refuge, and scours pools. In steep channels, wood creates additional roughness that helps to retain sediment and increase complexity at a unit scale. |
| Key wood density (KW/m) | Number of the largest wood pieces / length of survey reach (m). Threshold size for key wood classification based on average channel bed width (S2 Table) | Largest wood that is likely to have longer residence time and, therefore, a sustained effect on pool formation and deposition of spawning substrate. |
| ***Substrate Transport*** |  |  |
| *D_50_* | Median particle size from pebble count | Low *D_50_* suggests aggradation of fine sediment or large *D_50_,* scouring. A moderate *D_50_* may signal presence of gravel that is suitable substrate for salmon spawning and egg incubation. |
| Relative submergence (RelSub) | Mean bankfull depth / D_50_ [31] | Provides information about sediment transport dynamics in pools. Moderate values are considered reference conditions. |

**S2 Table.** Minimum size thresholds for wood pieces to be considered “key wood.” CBW- Channel Bed Width.

| Average CBW (m) | Key piece diameter (m) | Key piece stem length (m) | Rootwad diameter (m) |
| --- | --- | --- | --- |
| 0–4.9 | 0.3 | >3 | >1 |
| 5–9.9 | 0.3 | >7.6 | >3 |
| 10–19.9 | 0.6 | >7.6 | >3 |
| ≥20 | 0.6 | >15 | >3 |

**S3 Table.** Best-supported models for each variable. Refer to Tables 1 and 2 for variable abbreviations. “TF” specifies whether response variable was natural log transformed (LN) or not (“No”). N is the sample size of available surveys used in that model. MR^2^ is the marginal pseudo R^2^ representing the amount of variance explained by fixed variables and CR^2^ and the conditional pseudo R^2^ representing the amount of variance explained by the fixed and conditional variables. See table S1 and Table 1 for variable abbreviation explanations. See table S1 and Table 1 for variable abbreviation explanations.

| **Category** | **Top Model Variables** | **TF** | **N** | **df** | **MR^2^** | **CR^2^** |
| --- | --- | --- | --- | --- | --- | --- |
| Channel  morphology |  |  |  |  |  |  |
|  | UB~Site,Ws,Yr,Rest,BuffHarvMax | LN | 588 | 7 | 0.11 | 0.68 |
|  | WidDep~CatArea,PG,Site,Ws,PPHarvestPropPre,RDCrossDens | LN | 610 | 11 | 0.28 | 0.74 |
| Pools |  |  |  |  |  |  |
|  | Pools/KM~CatArea,PG,Site,Ws,Yr,Rest, | No | 453 | 8 | 0.16 | 0.66 |
|  | PoolSpace~CatArea,Site,Ws,Yr,Rest | LN | 453 | 7 | 0.09 | 0.77 |
|  | RPD~CatArea,PG,Site,Ws,Yr,Rest,RDLenDens | No | 763 | 12 | 0.19 | 0.93 |
|  | PoolSize~Site,Ws,Yr,RdCrossDens | LN | 558 | 6 | 0.03 | 0.51 |
| Wood |  |  |  |  |  |  |
|  | LW/m~PG,Site,Ws,Yr,HydroClass,BuffHarvMax,HarvLag,OldGrowth | LN | 784 | 14 | 0.10 | 0.63 |
|  | KW/m~CatArea,PG,Site,Ws,Yr,HydroClass,OldGrowth | LN | 766 | 12 | 0.11 | 0.57 |
| Substrate  transport |  |  |  |  |  |  |
|  | RelSub~CatArea,PG,Site,Ws,Yr,Rest,PPHarvMax | LN | 502 | 12 | 0.17 | 0.71 |
|  | D50~CatArea,PG,Site,Ws,Rest,RDCrossDens | No | 547 | 10 | 0.10 | 0.75 |

**S4 Table.** Descriptions of channel process group classes.

| **Channel process groups modified from Paustian (1992; 2010)** | **Description** |
| --- | --- |
| Alluvial fan | Alluvial fan channels may have a range of gradients but are commonly identified by their position at the transition from confined high-gradient channels draining mountainous subwatersheds to unconfined lower-gradient valleys. These characteristics allow coarse materials to be deposited as stream transport power abruptly drops. |
| Floodplain | Low-gradient <2% slope channels of various sizes with wide valleys and broad riparian areas that are commonly inundated during high flows. |
| High-gradient | High-gradient channels >6%. The Paustian classification breaks out high-gradient channels into multiple process groups based on channel incision and valley confinement, however we combined them due to limited sample size. |
| Moderate-gradient | Moderate-gradient, 2–6%, slope channels of various sizes and degrees of channel confinement. The Paustian classification breaks out moderate-gradient channels into multiple process groups based on valley confinement and stream size, however we combined them owing to limited sample size. |
| Low-gradient | Low-gradient, <2% slope, channels of various sizes that have less floodplain access and less well-defined riparian zones than floodplain channels. |

**S5 Table.** Multi Response Permutation Procedure (MRPP) pairwise comparisons for three management classes and two process groups. A is the effect size and P is the p- value for the test. The significance threshold was adjusted for multiple comparisons using Bonferroni correction 0.05/4 tests = 0.013.

| **Group Comparison** | **A** | **P** |
| --- | --- | --- |
| Harvested:Reference | 0.0043 | 0.0390 |
| Harvested:Restored | 0.0248 | 0.0001 |
| Restored:Reference | 0.0531 | <0.0001 |
| Floodplain:Moderate-gradient | 0.0433 | <0.0001 |
