# Supplementary figures and images for: Same streams in a different forest? Investigations of forest harvest legacies and future trajectories across 30 years of stream habitat monitoring on the Tongass National Forest, Alaska
Source: PLoS One. 2024 Jul 10;19(7):e0301723. doi: 10.1371/journal.pone.0301723 (PMC11236125; doi:10.1371/journal.pone.0301723)

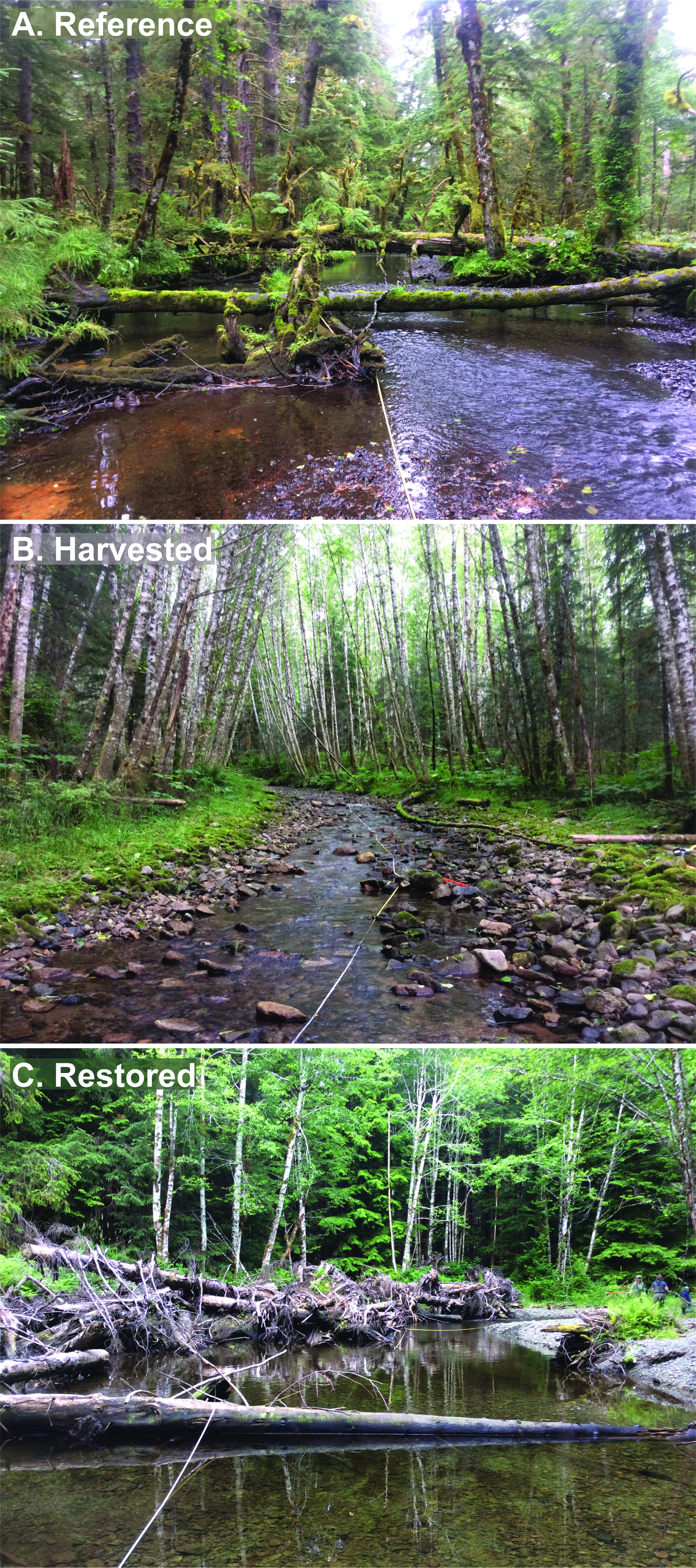

Supplement: S1 Fig — Panel A shows a stream in reference condition with no riparian buffer and limited subwatershed harvest. Panel B shows a site with substantial buffer and subwatershed harvest. Panel C shows a restored site that had buffer harvest and wood was installed to help improve stream habitat conditions. Collectively, these panels illustrate the multi-storied forest condition of reference stands, the conversion to Alder dominated riparian forest post-harvest that is discussed in the paper, and it shows some installed wood pieces at restoration sites. (TIF) [file pone.0301723.s002.tif]

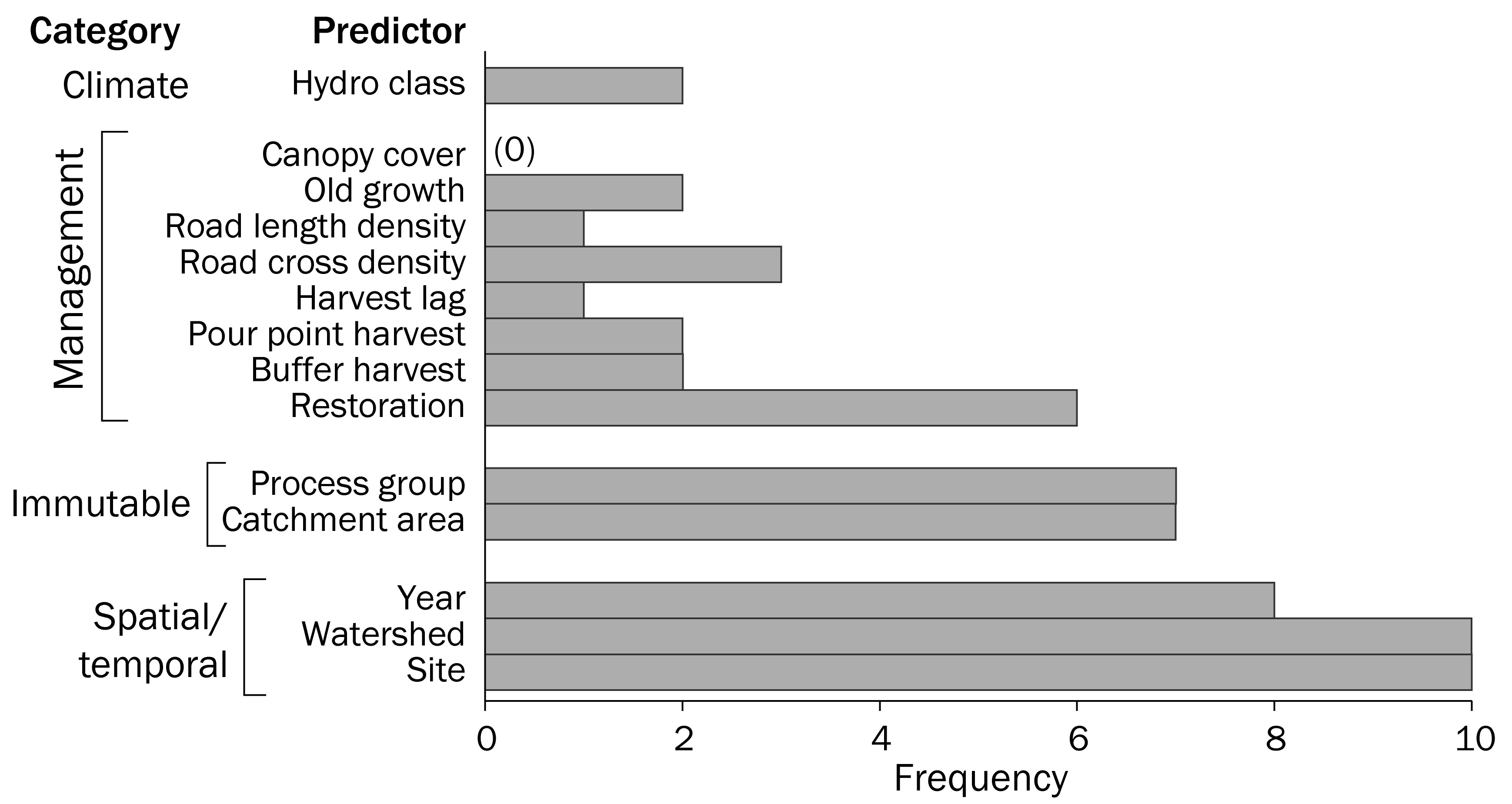

Supplement: S2 Fig — Bar position and x-axis labels divide predictors into 4 groups. Spatial/temporal random effects, intrinsic landscape fixed effects, management fixed effects, and climate fixed effects. (TIF) [file pone.0301723.s003.tif]

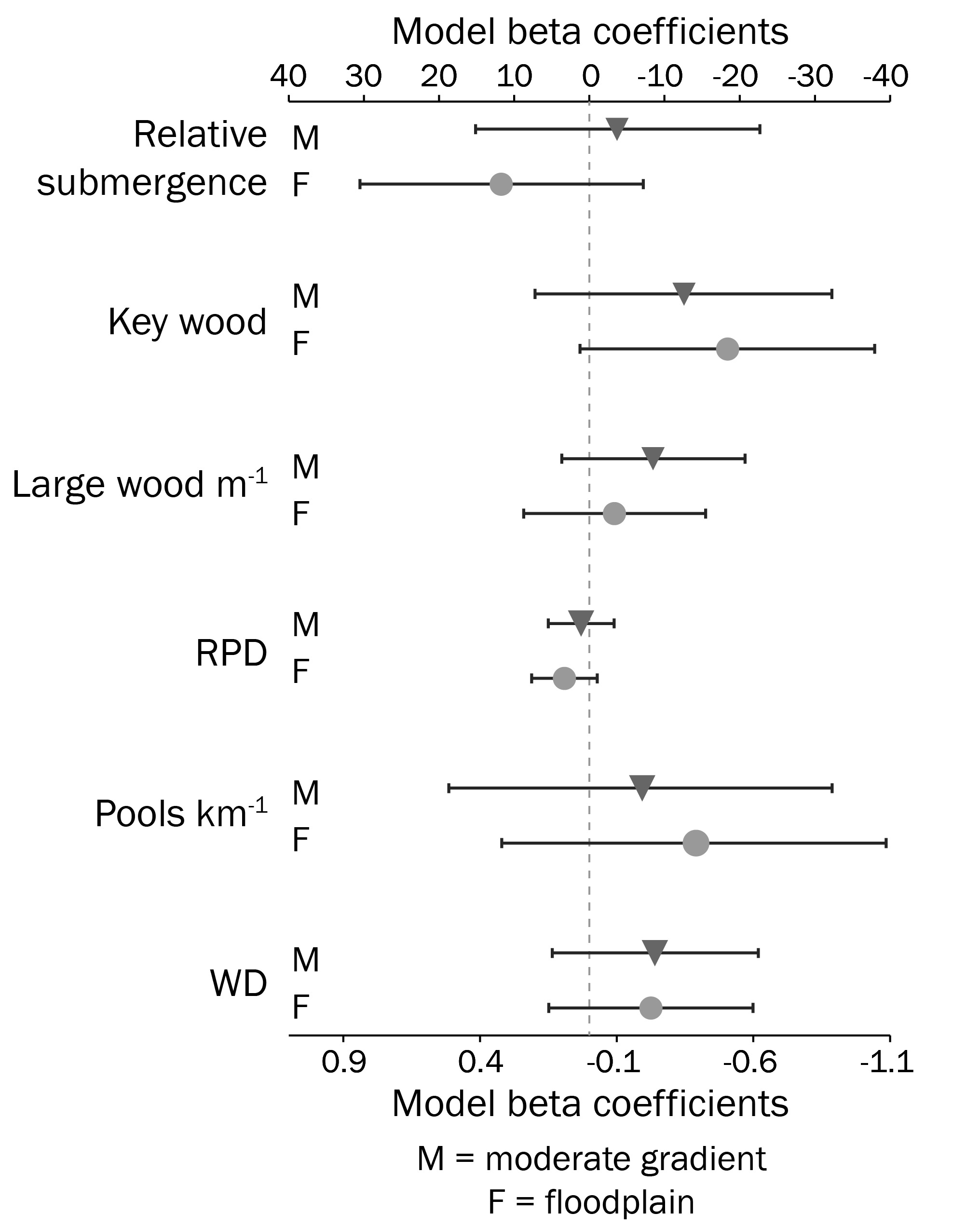

Supplement: S3 Fig — (TIF) [file pone.0301723.s004.tif]
