# Supplementary material for: Same streams in a different forest? Investigations of forest harvest legacies and future trajectories across 30 years of stream habitat monitoring on the Tongass National Forest, Alaska
Source: PLoS One. 2024 Jul 10;19(7):e0301723. doi: 10.1371/journal.pone.0301723 (PMC11236125; doi:10.1371/journal.pone.0301723)
Supplement: Attachment — Submitted filename: ADPManuscriptSubmissionPlosOne.pdf [file pone.0301723.s005.pdf]

This manuscript is distributed solely for purposes of peer review. Its content is deliberative and predecisional, so it must not be disclosed or released by reviewers. Because the manuscript has not yet been approved for publication by the US Geological Survey (USGS), it does not represent any official USGS finding or policy.

Same streams in a different forest? Investigations of forest harvest legacies and future trajectories across 30 years of stream habitat monitoring on the Tongass National Forest, Alaska

Moore, M. J. [1], Flitcroft, R. L.[2], Tucker, E.[3], Prussian, K. M. [4], Claeson, S. M.[5]

Short Title: Forest harvest legacies and future trajectories on stream habitat in the Tongass National Forest, Alaska

[1] U.S. Geological Survey, Iowa Cooperative Fish and Wildlife Research Unit, 339 Science II, 2310 Pammel Drive, Ames, IA, United States of America

[2] U.S. Forest Service, Pacific Northwest Research Station, Corvallis Forestry Sciences Lab, 3200 SW Jefferson Way, Corvallis, OR, United States of America

[3] U.S. Forest Service, Tongass National Forest, 123 Scow Bay Loop Road, Petersburg, AK, United States of America

[4] U.S. Forest Service, Tongass National Forest, 2108 Halibut Point Road, Sitka, AK., United States of America

[5] U.S. Forest Service, Pacific Northwest Research Station, Wenatchee Forestry Sciences Lab 1133 N Western Ave, Wenatchee, WA, United States of America

*Corresponding Author

E-mail: mooremj@iastate.edu (MM)

Abstract

The effects of timber harvest practices and climate change have altered forest ecosystems in southeast Alaska. However, quantification of patterns and trends in stream habitats associated with these forests is limited owing to a paucity of data available in remote watersheds. Here, we analyzed a 30-year dataset from southeast Alaska’s Tongass National Forest to understand how these factors shape stream habitats. First, we examined differences between broad management classes (i.e., harvested and non-harvested) that have been used to guide stream channel restoration goals. Second, we assessed associations between intrinsic landscape characteristics, watershed management, and timber harvest legacies on aquatic habitat metrics. And third, we examined trends in stream habitat metrics over the duration of the dataset to anticipate future management challenges for these systems. Small effect sizes for some harvest-related predictors suggest that some stream habitat metrics, such as pool densities, are less responsive than others ~~and management practices such as protecting riparian as well as post-harvest restoration may be helping~~, and management practices such as protecting riparian as well as post-harvest restoration may help conserve fish habitats. Large wood densities increased with time since harvest at sites harvested >50 years ago, suggesting that multiple decades of post-harvest forest regrowth may contribute large wood to streams (possibly alder), but that it is not enough time for old-growth trees (often spruce or hemlock), classified as key wood, to develop and be delivered to streams. The declining trend in key wood (i.e., the largest size class of wood), regardless of management history, may reflect that pre-harvest legacy old-growth trees are declining along streams, with low replacement. The introduction of wood to maintain complex stream habitats may fill this gap until riparian stands again contribute structural key wood to streams. Trend analyses indicate an

increasing spatial extent of undercut banks that may also be influenced by shifting hydrologic regimes under climate change.

Introduction

The Anthropocene is characterized by the signature of human alteration of the climate and landscape on a global scale [1], [2]. Changes in the composition and structure of remote forested watersheds at northern latitudes are likely to accelerate, reflecting increasing global demand for natural resources, accumulation of persistent pollutants, and climate models that project atmospheric warming and increased frequency of droughts and floods. Southeast Alaska is characterized by forested systems distributed across numerous island and mainland areas. This area is the largest intact temperate rainforest on earth and includes the 68,800-km^2 (17-million-acre) Tongass National Forest (NF), the largest NF in the United States (Fig 1A) [3]. Management of NFs support a regional socio-economic system reliant on interconnected renewable natural resources such as fisheries, freshwater, and forestry [4]. From 2007 to 2019, the Tongass NF produced 75% of Southeast Alaska's commercial salmon harvest, with an average 2019 dockside annual value of US $68.5 million dollars per year [5]. Species such as coho salmon (*Oncorhynchus kisutch*), whose parr will remain in freshwaters for up to two years, and resident freshwater species such as Dolly Varden (*Salvelinus malma*) and cutthroat trout (*Oncorhyncus clarkii*) are sensitive to stream channel alteration owing to forestry practices, and thus are considered indicators of aquatic ecosystem health [6]. The forested landscape these streams flow through is changing as a result of ongoing forest management as well as climate change (i.e., temperature and precipitation).

Fig 1. Paneled study area map. Panel A shows the location of each survey site and the location of the study area relative to the State of Alaska boundaries. The panels that follow depict the spatial distributions of 8 continuous variables through the scaling of survey-site point size. Catchment area is represented by a discrete graduated symbol-size scale. Continuous variables in other panels are represented by establishing a minimum point area for the smallest value of the variable and linearly increasing the point size proportional to that variable. Tongass NF boundaries are shaded green.

Comentado [A1]: Can you provide a map of the streams?

Comentado [A2]: Check: the hydro class is a categorical variable

Con formato: Centrado

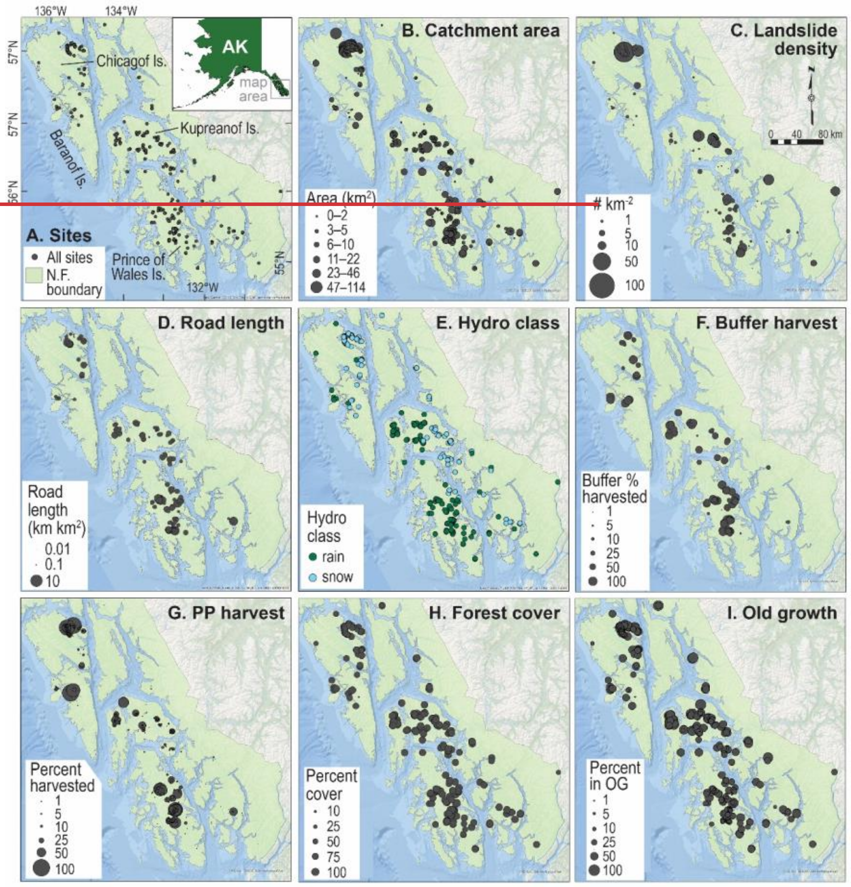

Research on the responses of stream habitats to forest management in the coastal temperate forests of Alaska and Canada have focused on the effects of timber harvest on stream geomorphology and stream discharge [7]–[10]**,** and the effects of harvest practices [10], [11], roads [12], large wood [13], [14], and sediment [15] on salmonid habitat. For example, logging roads can route overland runoff and fine sediments into stream channels, which may reduce salmon spawning-habitat suitability [12]. The supply of large wood pieces may be reduced post-harvest [16]. Wood is crucial for creating cover, capturing sediment, and forming low-velocity rearing pools for juvenile salmon and is dependent on riparian stand age and mortality rates [13], [14].

Teasing apart the effects of land-use legacies on habitat condition trajectories in a changing climate is challenging. Northern forests are predicted to change as a result of disturbances (e.g., invasive species, insects, disease, climate) that will alter tree growth, mortality, and phenology, with cascading consequences for terrestrial and aquatic biophysical systems [17], [18]. Climate change is predicted to affect river hydrologic regimes, which reflect patterns of precipitation and temperature. For instance, winter flow is lower in watersheds that receive more precipitation as snow as compared to rain-dominated systems, but snowmelt in these systems provides maintenance flows later into the summer than rain-dominated systems. Thus, as snow-dominated systems transition to rain-dominated systems, timing of water availability will also change, potentially disrupting phenological adaptation to environmental conditions for aquatic species and ecosystems [19]. Additionally, warmer temperatures may produce more intense winter rain in previously snow-dominated systems, that may lead to flooding and streambed scour, altering channel morphology, and causing increased salmonid egg mortality [20], [21].

The impacts on Alaskan salmon populations from timber harvest, climate change, and stream restoration will likely vary spatially [22] and be species-specific owing to differences in life-history traits and habitat requirements [23]. Therefore, coordinated monitoring efforts to support science-driven management recommendations, goals, and actions may be valuable in detecting complex effects. Assessment methods for evaluating aquatic habitat response to forest-harvest practices can be divided into three designs: 1) long-term studies of experimental manipulations of a single site; 2) control and impact monitoring studies; and 3) extensive survey programs of sites with diverse management histories that substitute spatial replication for temporal replication [24]. Historically, uniform timber-harvest practices, implemented under what was considered to be a stable climate, allowed the Tongass National Forest (NF) to monitor streams with standard, broad management classification frameworks that contrasted stream habitats in watersheds that had any level of harvest versus reference watersheds that had none. This aligns more with the control and impact paradigm. For example, Bryant et al. [7] noted significant differences between sites in harvested and non-harvested watersheds in terms of total wood and pool densities, residual pool depth, and median particle size in some geomorphic stream classes.

However, multiple harvests and changing practices over many decades have produced a complex mosaic of forest stand ages on the landscape. Intensive early harvest practices clearcut riparian zones and, in some cases, removed in-channel wood. In contrast, more recent (since the mid-1990s) harvest events are smaller in scale and have included riparian buffer protections. This variety of historical harvest practices and time since harvest among sites calls for a new assessment and monitoring approach. Assessment option 3 leverages newly available temporally and spatially expansive datasets collected using standardized habitat-assessment protocols.

Under this monitoring option, analysis of ~~fluvial~~ stream habitats on the Tongass NF would include statistical tools that control for variation to better explore the complex interacting consequences of changing timber-harvest management approaches, ~~as well as~~ stream restoration and climate change.

To facilitate ongoing adaptive management on the Tongass NF, we assessed stream habitat characteristics along a gradient of intrinsic characteristics and forest harvest practices using the most comprehensive long-term dataset assembled for southeast Alaska to-date. Analysis was completed following three sequential objectives. The first objective was to compare stream habitat conditions in harvested and unharvested watersheds on the Tongass NF using the historically applied control and impact classification framework. The second objective was to explore how gradients in landscape characteristics, legacies of forest management, and climate-driven hydrology may explain variation in stream habitat metrics on the Tongass using a more nuanced modeling approach to control for variation and disturbance magnitude. Finally, our third objective was to explore temporal trends in stream habitat metrics across the 30-year survey period to identify emerging management challenges. This work serves as a pathway to begin thinking of stream habitat characteristics in the Tongass along spatial and temporal harvest gradients similar to analyses that have been recently completed in other forested ecosystems of the Western US [25]–[27]. We hypothesize that habitat differences between sites with harvested and unharvested watersheds will have decreased over time owing to declining harvest intensity, forest regeneration, and protection of riparian buffers. We expect that the effects of specific harvest-related, climate, and restoration variables such as time since harvest, hydrologic regime, and active wood placement in streams may be better identified through the mixed-model regression approaches in objectives 2 and 3. The purpose of objectives 2 and 3 is to explore

Comentado [A3]: f there were information about beaver densities or dam densities, it would be constructive for the analyses.

correlative patterns in stream habitat conditions across space and time that may or may not match up with our expectations based on published literature. These patterns can serve as hypotheses to be examined with targeted, controlled research experiments in the future.

Materials and methods

Study site description

The study area has a coastal maritime climate characterized by cool temperatures and abundant rainfall (> 200 m~~c~~m y^{-1}), which ranges widely owing to marine influence and topography. Substantial snow occurs at higher elevations on islands of the Alexander Archipelago or on the mainland during the winter. Thus, narrow ranges in winter temperatures have substantial effects on stream hydrologic regimes [28]. The Tongass NF was established in 1907. The Tongass Timber Act of 1947 opened harvest for old-growth Sitka spruce (*Picea sitchensis*), western hemlock (*Tsuga heterophylla*), western redcedar (*Thuja* plicata), and Alaska cedar (*Xanthocyparis nootkatensis*). The first timber harvests following the Tongass Timber Act occurred between 1950 and 1969, and were concentrated in easily accessed, productive, lowland watersheds near the coast [6]. In addition to the aforementioned species, red alder (*Alnus rubra*) is prevalent in open areas disturbed by logging, floods, and landslides [29]. Regulations to protect stream habitats during logging were instituted in the Tongass Timber Reform Act of 1990, which mandated a 33-m-wide protected “riparian management buffer” (RMB) along fish-producing streams in harvested tracts [30]. Data collection for this study began shortly after the institution of this Act and continued until 2020. For our analyses, we also defined a “stream analysis buffer” (SAB) using GIS, extending out 91 m (250 ft) on either side of a surveyed site. This stream buffer is wider than the Tongass NF regulated buffer, but is similar to buffers

Comentado [A4]: check

afforded to streams on federal lands in other areas of the Pacific Northwest that are occupied by anadromous salmonids listed as threatened or endangered under the US federal Endangered Species Act [31]. Thus, the SAB is approximately 2.5 times the average tree height for 100-year-old trees [32] and is greater than the 33-m (100-ft) legally defined RMB width.

Stream habitat metrics relevant for sensitive aquatic biota

We used a dataset consisting of 852 habitat surveys from 1991 to 2020 at 323 fish-bearing stream reaches (hereafter, sites) that were predominantly accessible by road (some sites required helicopter or off-road access) (Fig 1A). ~~Sixty-three percent of the~~The sites were surveyed more than once, with the number of repeat surveys ranging from 1 to 13, with a mean of between 2 and 3 surveys/site. We delineated the upstream drainage subwatersheds using a digital elevation model with survey sites corresponding to the downstream pourpoints. The subwatersheds were nested within 98 HUC-12 level watersheds (hydrologic unit codes, United States Geological Survey, National Hydrography Dataset https://www.usgs.gov/core-science-systems/ngp/national-hydrography).

Comentado [A5]: Figure 1A does not show that information.

Comentado [A6]: Please add a map with relevant stream information for the manuscript´s goals, such as size, length, connectivity, and location.

The long-term habitat assessment database developed for this study comprises four major data-collection initiatives conducted as part of the Tongass National Forest Land and Resource Management Plan [33]; the Channel Condition Assessment (1996–1999, n = 28) [34]; general aquatic surveys (collected by USFS; 1991–2017, n = 91); Management Indicator Species monitoring studies[6], [35], and the Watershed Restoration Effectiveness Monitoring study (collected by USFS, 2013–2020, n = 146). This compilation of datasets resulted in a non-uniform temporal and spatial distribution across the study area over time. For example, 90% of the surveys were conducted after 2000 and 30% between 2012 and 2015.

We completed a preliminary assessment of survey data to ensure consistent survey protocols. Most surveys measured habitat metrics that are invariable with discharge, correlate with fish populations, and are commonly measured in traditional habitat surveys to assess physical stream characteristics over space and time (e.g., Tier II surveys) (Table 1) [36]. Not all metrics were measured in each stream survey, which depended on project-specific objectives. Sample sizes for individual metrics ranged from 453–784 surveys. The metrics analyzed in our multivariate and univariate statistical models and tests were associated with channel morphology (undercut bank density, width-to-depth ratio i.e., width:depth, pool density, pool spacing, residual pool depth, pool size, riffle area); large wood (large wood density and key wood density); or substrate transport (50th percentile particle size (D_{50}) and relative submergence) (Table 1).

Table 1. Stream habitat metrics that were used as response variables in multivariate and univariate statistical models and tests. Metrics are grouped into different categories (bold italic headings). Metric names are those used in the manuscript text; abbreviations in parentheses are used in tables and figures.

Stream habitat metric (abbreviation)	Description	Relevance to biota/ habitat condition
Channel mMorphology		
Hydraulic radius (radius)	Channel cross section area / cross section perimeter	General descriptor of channel cross-section shape. For a given cross-sectional area, relatively flat, shallow channels have a low radius, and deep, narrow channels have a high radius.
Width:depth (WD)	Mean bankfull width / mean bankfull depth	Measure of channel disturbance. Where there is channel widening or shallowing following floods/debris flows/loss of riparian vegetation, values will be high relative to reference conditions, but values will be low where channel entrenchment is occurring. Better condition produces intermediate values. Shallower depth can increase stream temperature.

Stream habitat metric (abbreviation)	Description	Relevance to biota/ habitat condition
Undercut bank density (UB)	Length of undercut banks / length of surveyed reach (m). Where vertical water depth is >0.3 m, measured as contiguous lengths separately for each bank and combined.	Undercut banks, where wetted at low to medium flow, are important cover habitat for fish. At high flows they provide refugia from center channel velocities. They are thought to be a sign of well-vegetated and intact banks.
Riffles		
Riffle area	Total area in riffle habitat	Riffles provide habitat for macroinvertebrate prey production and spawning habitat for salmonids.
Pools		
Pool density (pools/km)	Total number of pools / meters surveyed * 1000	Pools provide important rearing habitat for juvenile fishes, especially coho salmon which spend longer in freshwater as juveniles. Higher values are desirable because pools are usually more limited than salmon spawning habitat (i.e., riffles) in these streams.
Pool spacing (PoolSpace)	Average distance between pools expressed in units of channel width: (reach length / average width of channel bed from bottom of bank to bottom of bank) / number of pools [16].	See above. Lower values indicate less space between pools and thus higher pool density.
Relative pool area (RPA)	Surficial pool area / total stream area surveyed	Larger pool area = higher RPA. Pool area is important overwintering habitat for juvenile salmonids.
Residual pool depth (RPD)	Maximum pool depth minus pool tail (riffle crest) depth	Deeper pools may be more complex, have lower water velocities, and be less likely to freeze over winter, making them important fish habitats.
Residual pool depth/ channel bed width (RPD/CBW)	Residual pool depth (as defined above) normalized by bed width	See above; adjusts RPD for stream size.
Pool size (PoolSize)	Mean RPD / mean bankfull depth [31]	A measure of pool quality to describe the depth of pools relative to other stream features. Higher values are better because deep pool habitat is usually limited by lack of large wood at disturbed sites.
Wood		
Large wood density (LW/m)	Number of large wood pieces (>1 m in length and 0.1 m in diameter) within the bankfull channel / length of survey reach (m)	Large wood provides macroinvertebrate habitat, juvenile fish refuge, and scours pools. In steep channels, wood creates additional roughness that helps to retain sediment and increase complexity at a unit scale.
Key wood density (KW/m)	Number of the largest wood pieces / length of survey reach (m). Threshold size for key wood classification based on average channel bed width (S1 Table)	Largest wood that is likely to have longer residence time and, therefore, a sustained effect on pool formation and deposition of spawning substrate.
Substrate Transport		
D_{50}	Median particle size from pebble count	Low D_{50} suggests aggradation of fine sediment or large D_{50}, scouring. A moderate D_{50} may signal presence of gravel that is suitable substrate for salmon spawning and egg incubation.
Relative submergence (RelSub)	Mean bankfull depth / D_{50} [31]	Provides information about sediment transport dynamics in pools. Moderate values are considered reference conditions.

Categorical land management classification

Stream habitat assessments on the Tongass NF have been classified in the past based on harvest and land management history (i.e., no timber harvest versus harvest with or without riparian protections) as a simple way to facilitate stream restoration and to identify habitat metrics most influenced by timber harvest [7]. Therefore, we evaluated whether habitat differences found in previous analyses existed for the following three broad classes: 1) “reference” sites were identified as subwatersheds with <6% harvest area and no harvest within the SAB; 2) “harvested” sites had >6% subwatershed harvest area or some amount of SAB harvest; and “restored” sites which had past harvest but also had experienced restoration primarily through the installation of large or key wood pieces in the stream prior to site surveys.

Landscape template as habitat predictors

Controlling for the natural variability in landscape characteristics allows us to better isolate management and climate effects on channel morphology and aquatic habitat [34]. Therefore, we compiled data from several GIS layers that represent intrinsic physiographic characteristics of subwatersheds (Table 2).

Table 2. Candidate predictors in linear mixed models, LMMs. Predictor names are those used in the manuscript text; abbreviations in parentheses are used in tables and figures.

Predictor (abbreviation)	Description	Data source
Random Effects		
Watershed (WS)	HUC12 watershed name to account for spatial nesting of sites within watersheds	National Hydrography Dataset https://www.usgs.gov/core-science-systems/ngp/national-hydrography

Predictor (abbreviation)	Description	Data source
Site	Site identifier to account for correlation attributable to repeat surveys at sites.	
Year (YR)	The year a survey was conducted to account for temporal autocorrelation	
Intrinsic		
Process Group (PG)	Stream channel classification based on hydrologic function, landform, and channel morphology. Classes combined into moderate gradient, floodplain, alluvial, and high gradient.	Paustianet al. 1992
Subwatershed Area (CatArea)	Natural log of subwatershed drainage area (km^2) using downstream site boundary as a pourpoint	Digital elevation model (DEM). 1-m LiDAR DEM for Prince of Wales Island, 5-m IfSAR DEM for rest of study area
Management		
Old Growth Forest (OldGrowth)	Proportion of the subwatershed area in size class 4 forest (>22.9 cm [>9 in] diameter at breast height or 137 cm [4.5 ft] above the ground) and defined as "old-growth sawtimber" >150 years old	Covertype layer USFS Region 10
Canopy Cover (Canopy)	Proportion of the subwatershed area in any type of forest.	Covertype layer USFS Region 10
Pourpoint Subwatershed Harvest (PPHarv)	Proportion of subwatershed area that had been harvested prior to the habitat survey date	Combination of harvest data from USFS and other landowners, such as State of Alaska, Sea Alaska Corporation, and Alaska Department of Natural Resources
Riparian Harvest (RipHarv)	Proportion of a reach's stream analysis buffer (SAB, 91 m wide on each side of the stream) harvested prior to the habitat survey date	Overlap of harvest data (see above) with 91-m stream analysis buffer applied on each side of surveyed stream reach.
Harvest Lag (HarvLag)	Number of years between the date of the largest buffer or pourpoint harvest event for a given site and the date of that survey	
Road Crossings (RDCrossDens)	Number of intersections of road and stream layer files in a subwatershed divided by subwatershed area	System roads from the Tongass National Forest Transportation Database and AKHydro Stream Lines.
Road Lengths (RDLenDens)	Length of road in a subwatershed divided by subwatershed area	USFS Tongass NF Transportation database - System Roads
Restored (Rest)	Binary variable for whether restoration in the form of large wood addition occurred prior to a survey. Over 99% of restoration actions were restoring large wood to stream channels	
Climate		
Hydrologic Regime (HydroClass)	Broad scale hydrologic classifications for each survey site based on dominant precipitation type: rain (includes transitional rain–snow regimes) vs. snow	Sergeant et al. 2020.

Channel gradient and confinement were accounted for with a categorical variable based on region-specific channel process groups that are used extensively by land managers on the

Tongass NF. Owing to the limited sample size (see Table 3 for model-specific sample sizes), we combined some of the process groups defined in Paustian et al. [37] based on gradient. For example, all moderate-gradient process groups were combined into a single group. Final process groups used in our dataset include alluvial fan, floodplain, and low-, moderate-, and high-gradient reaches. We focus primarily on floodplain and moderate-gradient process groups because they are responsive to disturbances, generally contain productive forests that were targeted for historic harvest, and reflect the combined influences of habitat changes throughout upstream reaches of the stream network (S2 Table) [34].

Table 3. Best-supported models for each variable. Refer to Tables 1 and 2 for variable abbreviations. "TF" specifies whether response variable was natural log transformed (LN) or not ("No"). N is the sample size of available surveys used in that model. MR^2 is the marginal pseudo R^2 representing the amount of variance explained by fixed variables and CR^2 and the conditional pseudo R^2 representing the amount of variance explained by the fixed and conditional variables.

Category	Top Model Variables	TF	N	df	MR^2	CR^2
Channel m~~M~~orphology						
	UB~Site,Ws,Yr,Rest,BuffHarvMax	LN	588	7	0.11	0.68
	WidDep~CatArea,PG,Site,Ws,PPHarvestPropPre,RDCrossDens	LN	610	11	0.28	0.74
Pools						
	Pools/KM~CatArea,Site,Ws,Yr,Rest,BuffHarvPropPre	No	453	8	0.16	0.66
	PoolSpace~CatArea,Site,Ws,Yr,Rest	LN	453	7	0.09	0.77
	RPD~CatArea,PG,Site,Ws,Yr,Rest,RDLenDens	No	763	12	0.19	0.93
	PoolSize~Site,Ws,Yr,RdCrossDens	LN	558	6	0.03	0.51
Wood						
	LW/m~PG,Site,Ws,Yr,HydroClass,BuffHarvMax,HarvLag,OldGrowth	LN	784	14	0.10	0.63
	KW/m~CatArea,PG,Site,Ws,Yr,HydroClass,OldGrowth	LN	766	12	0.11	0.57
Substrate ~~Transport~~transport						
	RelSub~CatArea,PG,Site,Ws,Yr,Rest,PPHarvMax	LN	502	12	0.17	0.71
	D50~CatArea,PG,Site,Ws,Rest,RDCrossDens	No	547	10	0.10	0.75

Forest Management

Disturbances can drive eco-physical responses at multiple scales, making multi-scale analyses important in understanding ecological processes [38]. Therefore, we consider the effects of harvest at multiple spatial scales by calculating the amount of prior cumulative forest harvest a~~s~~ ~~well as~~nd the largest harvest event for each survey at the subwatershed scale and within the SAB. Stream habitats respond in a variety of ways to timber harvest in the upstream subwatershed and in the riparian area surrounding each sample site, including persistent shifts in mean values or trends over time [39]. Therefore, we included a lag metric for time since harvest that could interact with the amount of harvest at the subwatershed and SAB extents. We also considered a quadratic relationship with harvest lag based on the hypothesis that remnant slash and riparian windthrow may increase wood densities during episodic events after harvest but later gradually decline owing to reduced supply from regenerating forests.

In addition to forest harvest, we used the most recent GIS layer for forest-stand condition, provided by the Tongass NF, to calculate the area of productive old-growth forest (i.e., the proportion of a watershed with trees estimated at >23 cm (9 in) dbh and over 150 years in age) and canopy cover (i.e., forest of any kind) at the subwatershed scale. We also summarized the length of roads and the number of road crossings at the subwatershed scale. Instream restoration (usually the addition of large or key wood pieces) has been completed at many sites across the Tongass NF. Exploratory analyses in objective 1 found that stream habitat at restored sites could differ from non-restored sites. Therefore, we evaluated a binary variable for whether a site had experienced in-stream restoration (i.e., wood restoration prior to the survey date for objective 2; we excluded restored sites from the trend analyses in objective 3).

Climate Data

To capture differences in stream habitat that could be related to hydrologic regimes, we used hydrologic classifications of rain-dominated and snow-dominated [28]. Watersheds characterized as transitional between rain- and snow-dominated were grouped with the rain-dominated class owing to low sample size. The full dataset contained 514 sites with rain-dominated hydrology and 338 sites with snow-dominated hydrology. All predictor variable descriptions are provided in Table 2.

Statistical analyses

Multivariate tests of stream habitat differences in harvested, not harvested, and restored subwatersheds under the control and impact management classification framework

In objective 1, we used multivariate analyses to test the hypothesis that stream-habitat conditions on the Tongass NF were significantly different among the broad management classes of “reference”, “restored”, and “harvested”, as well as the floodplain and moderate-gradient process groups. We tested for differences in multivariate mean habitat conditions among management groups and channel-process groups using non-parametric, multi-response, permutation procedure (MRPP) simulation tests utilizing a Euclidian distance metric (R Vegan package, 999 permutations) [40]. Post-hoc pairwise comparisons between groups were conducted with the same method as multi-way comparisons, except that only two groups were tested at a time. We used the Bonferroni correction to adjust the significance level (α) for multiple comparisons. MRPP provides a p-value and the chance-corrected, within-group, agreement statistic (A) as an

effect size that ranges from −1 to 1, with values closer to 1 denoting greater intergroup difference. The effect size is important for interpreting the ecological significance of the test because sample sizes >100 can produce significant p-values with small effect sizes (i.e., A < 0.1) [41].

Habitat metrics included in our multivariate analysis were hydraulic radius, undercut bank density, width:depth, relative submergence, D_{50}, large wood density, key wood density, pool density, residual pool depth, residual pool depth/channel bedwidth, pool spacing, relative pool area, pool size, total pool area, and total riffle area. Multivariate analyses require complete data matrices, therefore we filtered out incomplete records leaving 346 surveys. We imputed missing values for undercut bank density for 7 survey records using the value from another survey of the same site that was closest in time. We overlaid group membership on a PCA ordination with metric loadings and site scores that graphically represented correlations of individual metrics with PCA axes and differences among sites in management classes and channel-process grouping.

Linear mixed models to elucidate roles of intrinsic landscape characteristics, management, and climate-driven hydrology on stream habitats

For objective 2, we used univariate linear mixed effects models (LMMs) to explore how more complex relationships among intrinsic landscape characteristics, management actions, and climate-driven hydrology may affect stream-habitat metrics (see Table 3 for model-specific sample size, *n*). We adapted stepwise modeling frameworks (e.g., Dunn et al. [42]) that have also been used to isolate the signatures of natural physiographic processes and the legacy of human disturbance in stream habitats in the Pacific Northwest [26], [27] into our own 6-step modeling

process conducted in the Lme4 package in R (Fig 2) [43]. All continuous predictors were centered and scaled by mean and standard deviation.

Fig 2. The 6-step univariate linear mixed model process. Abbreviations: rd (road), pp (pourpoint catchment), ln (natural log), cat. (categorical variable), cont. (continuous variable).

In step one (see Fig 2), we examined the distribution of the response variable for departures from normality. Transformations such as natural log were implemented when necessary to better approximate normality (i.e., skewness <|1|). A small constant was added to all response variable values when zero values were present prior to log-transformation.

In step two (see Fig 2), we accounted for nuisance spatial and temporal correlation of surveys and repeated measures of sites with random intercepts for the following factors: site, HUC 12 watershed, and survey year. We selected the random-effects structure based on minimum AIC [44] among all possible combinations of these three variables. We screened models for lingering ~~effects of spatial and temporal autocorrelation~~spatial and temporal autocorrelation effects by examining semivariograms.

In step three, we developed a base model to control for variation in the habitat metrics resulting from geomorphic processes and physiographic patterns. We started with a global model containing 4 intrinsic factors and 3 random effects listed previously (Table 2) that set a template to constrain ranges of variability in stream habitat [45]. Next, we eliminated non-informative predictors by backward-stepwise model selection using maximum likelihood methods that are required when comparing likelihood among models with different fixed effects [46]. In this process, we began with a global model containing all intrinsic variables, which was compared to

the suite of models in which a single variable was dropped. AIC was calculated for each model and then the least-supported variable was dropped and removed from further steps. The process repeated until no more variable removals were supported according to minimum AIC.

In step four, we added management variables in five categories: timber harvest, roads, landcover, hydrological classification, and restoration history (Fig 2). Hydrologic classification was included with other management variables because of the role of anthropogenic activities such as greenhouse gas emissions in driving ongoing climate change across the Tongass NF. Variables were added individually and the top supported variable based on minimum AIC was retained from each category for use in step five.

In step five, we combined the base intrinsic landscape model and added the top management/climate predictors from all categories selected in step four. We screened variable pairs for collinearity by identifying those with variance inflation factors >5 and pairs of variables with pairwise Pearson correlation coefficients >0.7, and removed the least supported of the pair. Next, we performed the same backwards selection procedure from step two on management and climate variables only.

In step six, the final model was refit using restricted maximum likelihood REML procedures that produce unbiased estimates of variance components [39], [46]. The parameter coefficients from the top models for each response metric were assessed individually to calculate effect sizes and determine relationships between management actions, climate, and stream habitat. We report conditional and marginal R^2 for the final model, which measures the amount of variation explained by random- and fixed effects, respectively [47], as well as partial R^2 for select predictors using the partR2 package in R [48].

A space-for-time analysis of temporal habitat trends across the Tongass NF

For objective 3, we examined potential trajectories in stream habitat conditions by isolating the effect of a temporal variable (i.e., survey year). Because of the low number of repeated surveys within stream reaches, we implemented a space-for-time approach, while also controlling for spatial variation explained by natural landscape factors. The lack of site-selection randomization could have biased temporal trends if we were unable to control for confounding trends in site-selection criteria over time. Therefore, we decided to only examine trends for undercut bank density, large wood density, and key wood density. These metrics were measured during many of the same surveys, and studies suggest observer measurement precision is sufficient for these variables to detect modest change over time and space [49]. We divided sites into reference and harvested management classifications introduced in objective 1 and examined whether trends vary by hydrologic regime. Thus, rain- and snow-dominated hydrology could be captured at the subwatershed scale, as these hydrologic regimes may be changing differently in response to interactions between management and climate change. Restored sites were excluded from these analyses owing to low sample size. All possible combinations between the additive and interactive effects of year and hydrology were fit and the top model based on minimum AIC was retained for inference. We are not aware of any systematic temporal biases in site-selection criteria, selected site condition, or habitat survey training over time, but these factors have the potential to affect our results if present. Furthermore, we attempted to control for nuisance variation ~~through the use of random spatial and fixed physiographic effects and by~~by using random spatial and fixed physiographic effects and separately analyzing trends in reference sites and harvested sites.

Results

Assessment of stream habitat differences in harvested, not harvested, and restored catchments under the control-impact management classification framework

The MRPP analyses found significant mean differences among management classes (harvested, not harvested, and harvested then restored) and process groups (floodplain and moderate gradient). However, in pairwise comparisons of management classes, between-group differences had very small effect sizes (mean A = 0.027) [41]. Furthermore, only the restored sites were significantly different from the other two management classes, with no significant difference observed between harvested and reference classes after implementing the Bonferroni correction to α for multiple tests (Table 4). However, the MRPP analysis suggested slightly stronger differences existed between floodplain and moderate-gradient sites owing to the landscape level factors captured in our modification of Paustian’s hierarchical channel process group system ($A = 0.04$ $p < 0.0001$).

Table 4. MRPP pairwise comparisons for three management classes and two process groups. A is the effect size and P is the ~~P~~ p-value for the test. The significance threshold was adjusted for multiple comparisons using Bonferroni correction 0.05/4 tests = 0.013.

Group Comparison	A	P
Harvested:Reference	0.0043	0.0390
Harvested:Restored	0.0248	0.0001
Restored:Reference	0.0531	<0.0001
Floodplain:Moderate-gradient	0.0433	<0.0001

In the PCA, the first two principle components collectively explained 45.2% of total variance. Axis 1 was positively correlated with key wood and pool densities (i.e., ~~more~~ shorter pools) and negatively correlated with residual pool depth and pool area (i.e., fewer but longer pools) (Fig 3A). Axis 2 was positively correlated with D_{50} and riffle area and negatively correlated with relative submergence (Fig 3A). There was a substantial overlap of the distribution of survey scores for the first two principle components among the three management classes, corroborating the small effect sizes from the MRPP (Fig 3B). The minimum convex polygons (defined from the outer edges of the data points in each group) for harvested sites were slightly larger than the other groups (Fig 3B), potentially suggesting that the variability in disturbance histories for harvested sites resulted in greater habitat variability relative to reference sites. PCA Axis 1 explained much of the difference between the floodplain and moderate-gradient process groups. Floodplain process groups were associated with greater residual pool depth, whereas moderate-gradient channels were associated with higher densities of key wood and pools (Fig 3C). The outer boundary of the minimum convex polygon for harvested sites

extended further to the right on Axis 1 (Fig 3B), a pattern driven primarily by some small floodplain and moderate-gradient harvested sites that had greater densities of key wood, greater pool density, and larger pool area (Fig 3D).

Fig 3. PCA biplots of 346 sites with full data matrices for all included metrics: A) vector loadings of individual metrics to show correlations along axes 1 and 2; and individual sites plotted by their eigenvalues for axis 1 and axis 2 with sites color coded by B) management class, C) process group, and D) management class and relative channel size (small or medium/large), which is a part of the Paustian [35] classification system.

Comentado [A7]: For PCA A, please include the sample and environmental variables that were considered. In other words, in addition to vectors, include the points (sites).

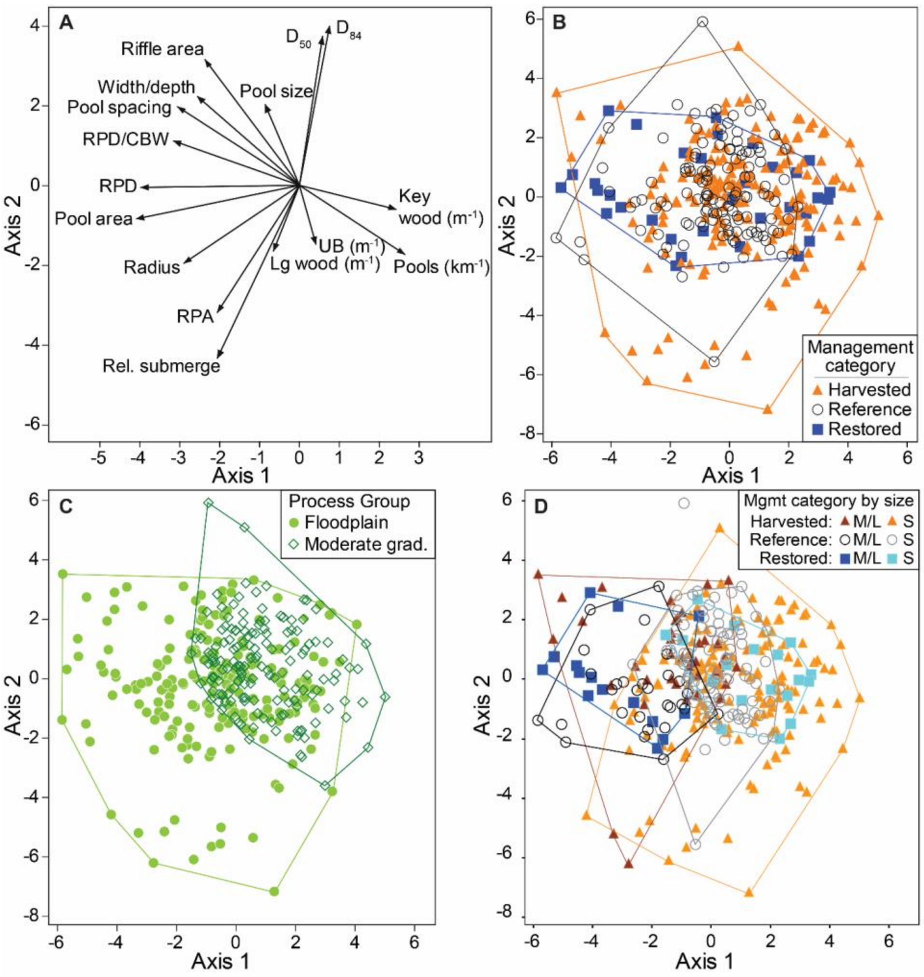

Variation in individual habitat metrics attributed to spatial, temporal, and intrinsic landscape factors

Spatially clustered patterns of geology and topography were apparent across the Tongass NF, with more similar characteristics for sites within watersheds. The proportion of the watershed classified as forest cover was high and relatively invariable across the study area (Fig 1H), reflecting regrowth of harvested areas. Most subwatersheds had high proportional area of forest cover (M = 92%, SD = 12.1), however, more variation existed in the proportion of old-growth forest cover (M = 42%, SD = 21.6), reflecting that past timber harvest has reduced the amount of old-growth forest (Fig 1F, 1G, 1H, 1I). Historical timber harvest at both the subwatershed and riparian-zone scales was concentrated in easily accessible coastal subwatersheds throughout Prince of Wales and Kupreanof islands, or near settlements of Baranof and Chichagof islands (Fig 1F; Fig 1G). Timber harvest was low for most mainland subwatersheds. The hydrologic influence of rain was greater in western lowland maritime-influenced subwatersheds and snow influence was greater in high-elevation island and eastern subwatersheds (Fig 1E). Road-length density had a similar spatial distribution as subwatershed timber harvest (r = 0.71) because most were timber harvest access roads. However, they were not simultaneously supported by model selection procedure and therefore never occurred in the same model.

Comentado [A8]: check

Random effects controlled for extra variation attributable to repeat surveys of sites (10/10 models), surveys of sites nested within HUC 12 watersheds (10/10 models), and surveys nested within years (8/10 models) (S1 Fig). Variation explained by fixed effects of best-supported models was low (marginal R^2 = 0.04–0.29) relative to random effects (conditional R^2 = 0.51–0.93; Table 3). The greatest amount of variation in habitat metrics was explained by fixed intrinsic and management predictors for width:depth (marginal R^2 = 0.28) and residual pool depth (marginal R^2 = 0.19). In many models, subwatershed area explained the greatest proportion of variation among fixed predictors and on its own explained ~50% of the fixed effect variation

in width:depth (marginal $pR^2 = 0.14$). In contrast, fixed predictors explained little variation in pool size (marginal $R^2 = 0.03$) or pool spacing (marginal $R^2 = 0.09$).

As expected, variation in stream habitat metrics was commonly associated with subwatershed area and process group (7 and 6 of 10 habitat metrics, respectively)~~, S~~; subwatershed area ranged from < 0.1 to 114.5 km^2 (Fig 1B) and had the largest effect size of any variable, with a 76% predicted increase in width:depth for a 25% increase in the observed range of subwatershed area (ln-transformed) (Table 5). Greater subwatershed area was also associated with greater pool spacing, residual pool depth, and relative submergence and lower pool- and key wood density. The overlapping 95% confidence intervals of beta coefficients for the floodplain and moderate-gradient group terms, which comprised a majority of the dataset, suggests that they were not statistically different (S2 Fig). Nonetheless, AIC-based model selection supported the inclusion of process group in several models, evidence that this variable may control for variation in stream habitat metrics, especially for process groups with lower sample sizes such as in the alluvial and high-gradient process groups.

Table 5. The coefficients (Beta), P values (*P*), and predicted marginal effect sizes (% change) of continuous or binary explanatory variables. The unit column reports whether the marginal effect size presented was for a 25% change in the observed range of a continuous predictor variable or a change from one level to the other of a binary variable. The change intervals (Lower; "LPI") and (upper "UPI") are 95% bootstrapped prediction intervals accounting for random effects and thus are wider than confidence intervals for the mean response. Refer to Tables 1 and 2 for variable abbreviations.

Response	Predictor	Unit	Beta	*P*	Change	Change 95 LPI	Change 95 UPI
UB	PostRest	Bin	-0.31	<0.001	−31.2%	−53.8%	−8.9%
	BuffHarvMax	0.25	-0.12	<0.001	−8.9%	−26.9%	7.8%
Width:depth	CatArea	0.25	0.28	<0.001	75.8%	25%	150.7%
	PPHarvestPropPre	0.25	0.06	0.086	6.2%	−12.8%	28.3%
	RdCrossDens	0.25	-0.06	0.047	−9.3%	−27.9%	19.1%
Pools/km	CatArea	0.25	-11.49	<0.001	−21.52	−38.24	−5.29
	PostRest	Bin	-11.14	0.037	−11.14	−23.66	1.76
	BuffHarvPropPre	Bin	-2.95	0.13	−1.87	−11.71	7.41
PoolSpace	CatArea	0.25	0.25	<0.001	60.2%	−16.7%	197.3%
	PostRest	0.25	0.75	<0.001	111.9%	38.5%	238.9%
RPD	CatArea	0.25	0.09	<0.001	18.5%	4.0%	33.0%
	PostRest	Bin	0.05	0.037	5.2%	−4.6%	15.4%
	RDLenDens	0.25	-0.02	0.037	−2.5%	−11.7%	8.2%
PoolSize	RdCrossDens	0.25	-0.07	0.008	−10.8%	−31.7%	9.7%
LW/m	HydroClass	Bin	0.13	0.033	16.5%	−0.8%	30.2%
	OldGrowth	0.25	0.05	0.041	6.5%	−7.1%	20.9%
KW/m	CatArea	0.25	-0.15	0.000	−24.5%	−50.2%	20.6%
	HydroClass	0.25	0.23	0.048	25.3%	1.6%	55.8%
	OldGrowth	0.25	0.08	0.065	10.1%	−10.7%	38.3%
RelSub	CatArea	0.25	0.10	0.007	22.5%	−24.8%	70.0%
	PostRest	Bin	0.20	0.067	20.4%	−6.5%	45.9%
	PPHarvMax	0.25	0.10	0.047	12.7%	−22.4%	43.9%
D50	CatArea	0.25	2.75	<0.001	5.68	−3.31	14.22
	PostRest	Bin	-3.62	0.14	−3.62	−8.68	−1.73
	RDCrossDens	0.25	-1.28	0.15	−5.48	−13.13	2.40

Variation in habitat metrics attributed to management legacies

Best-supported models for pool-metrics and D_{50} included few management variable effects (Tables 4 and& 5). In contrast, best-supported models for large or key wood densities contained

forest management predictors such as prior riparian harvest and proportion of old-growth cover within the subwatershed. However, these fixed effects had relatively small effect sizes (Table 5). Channel morphology metrics and relative submergence were explained by fixed effects from multiple predictor groupsFixed effects from multiple predictor groups explained channel morphology metrics and relative submergence.

The most consistently influential management predictor was restoration, which was included in best-supported models for 6 of 10 habitat metrics (Supplemental Table 1). Restored sites had higher pool spacing, lower densities of undercut banks and poolundercut banks and pools densities, lower residual pool depth, and lower D_{50}. In contrast, there were no differences in large and key wood densities based on restoration status.

Effects of harvest variables were noted at the subwatershed scale for two metrics and at the SAB scale for two other metrics. Harvest at the subwatershed scale was positively correlated with width:depth and relative submergence (i.e., channel widening, Table 5). SAB harvest was negatively correlated with undercut bank density. There was a complex relationship between large wood density and the interaction between proportional riparian harvest and a quadratic term for time since harvest. The model predicted higher large wood densities at sites with large riparian harvests that occurred >40 years prior to the survey compared with sites without riparian harvest or with recent riparian harvest (Fig 4).

Fig 4. Predictions of the marginal effects of the interaction between the largest proportional riparian harvest and a second-order term for years since harvest on the response variable large wood density (large wood per length of surveyed reach in meters). Relationships are illustrated for three different time-spans of harvest lag, namely, 10, 35, and 70 years post-harvest. The raw survey data are displayed by the points with color representing the time

between harvest and survey years. The figure illustrates no predicted change in large wood densities in surveys ≤35 years post-riparian harvest for any magnitude of riparian harvest. However, sites surveyed >60 years following a large riparian harvest had higher predicted densities of large wood.

Proportion of subwatersheds in old-growth forest cover is another sign of lack of recent harvest, and was primarily associated with wood-related metrics. Both large wood and key wood densities increased with increasing proportions of old-growth forest. No relationships were identified between subwatershed canopy cover and habitat metrics.

Road-crossing density was negatively associated with pool size. Road-crossing density and road density were retained in models for width:depth and residual pool depth, respectively. However, the effect sizes were small, with prediction intervals that were nearly symmetric around 0 (Table 5).

Sites with a rain- or snow-dominated subwatershed hydrology did not differ for metrics describing channel morphology and sediment transport that are often predicted to respond to hydrologic variability. Instead, sites in snow-dominated subwatersheds had 16.5% greater large wood densities and 25% greater key wood densities than those in rain-dominated subwatersheds (Table 5).

Space for time analysis of stream habitat trends across the Tongass NF

Sample sizes of surveyed sites varied across habitat metrics in the reference, harvested, and combined datasets, which influenced statistical power available to detect temporal trends. Key wood ($N_{combined}$ = 784, $N_{reference}$ = 370, $N_{harvested}$ = 343) and large wood ($N_{combined}$ = 766, $N_{reference}$ = 357, $N_{harvested}$ = 338) had the largest sample sizes and 98% of the measurements of large wood density had a paired measurement of key wood density in the same survey. In contrast, most other metrics had data spanning the period from approximately 1996 to 2020 (Fig 5), however undercut banks were measured at few harvested site surveys prior to 2010.

Fig 5. Predicted time trends for three response variables, with raw data points, and trend lines are for rain (red), snow (blue), or combined (black) hydrologic classifications. ~~Where a difference between rain and snow hydrologies was not supported for that dataset and response variable combination, plots are in grayscale~~Plots are in grayscale where a difference between rain and snow hydrologies was not supported for that dataset and response variable combination. Plot rows are organized by response variable, with panels A., B, and C displaying the predicted marginal effect of year on undercut banks m^{-1} by year, plots D, E, and F the marginal effect of year on large wood m^{-1}, and plots G, H, and I the marginal effect of year key wood m^{-1}. The columns represent the dataset used in the model. The first column (combined) was fit using all available habitat surveys (including restoration sites with wood additions), the second uses only reference sites, which are defined as having <6% pourpoint subwatershed harvest, and the third column (harvested) uses all sites with >6% pourpoint subwatershed harvest regardless of whether the harvest was riparian or buffered, but excluding restored sites that had pre-survey wood additions.

Year was retained in all 9 of the best-supported models (3 metrics x 3 datasets), showing support for temporal trends (Table 6). Interactions between the hydrologic regime and year variables allow trend slopes to differ for rain- versus snow-dominated sites and were supported in 4 of 9 models (Table 6). Temporal patterns were generally similar among analyses conducted with the combined, reference, and harvested datasets. Notably, there were decreasing densities of key wood in all datasets except for snow-dominated sites in the harvested dataset.

Table 6. Marginal effect sizes and their 95% bootstrapped prediction intervals of a 10-year change in survey year for five habitat metrics. Hydroclass denotes rain- or snow-dominated hydrologic regimes. Where predicted effects are the same for both rain and snow, the model selection supported models with no interaction between hydroclass and year predictors. The landscape predictors column shows the natural landscape-level variables that were used to control for nuisance variation in response attributable to management or climate changes over time. See Tables 1 and 2 for variable abbreviations. Positive relationships are in bold face, negative relationships are in italics.

**Response**	**Landscape predictors**	**Hydro class**	**Combined**	**Reference**	**Harvested**
UB	None	Rain	**49.4 (29.2–72.4)%**	**21.6 (12.3–40.9)%**	**97.1 (56.0–147.7)%**
		Snow	**26.2 (10.3–45.3)%**	**21.6 (12.3–40.9)%**	**97.1 (56.0–147.7)%**
LW m^{-1}	PG+	Rain	**26.2 (15.6–37.1)%**	**26.6 (13.0–41.1)%**	**33.9 (15.7–55.0)%**
		Snow	**13.0 (1.2–24.7)%**	**7.0 (-6.2–21.8)%**	**33.9 (15.7–55.0)%**
KW m^{-1}	CatAreaKM+PG+	Rain	−13.5 (−26.3– 2.8)%	*−25.3 (−3.9– −42.1)%*	−14.0 (−30.2–6.2)%
		Snow	−13.5 (−26.3– 2.8)%	*−25.3 (−3.9– −42.1)%*	**42.7 (5.7–90.3)%**

The direction of metric trends differed between rain- and snow-dominated hydrologic regimes in only 1 of 9 best-supported models. Predicted effect sizes from best-supported models

include increases in undercut bank densities of 22 to 97% over 10 years for the reference, harvested, and combined datasets. Contrasting trends emerged for large wood and key wood densities in the reference and combined datasets. Large wood densities increased between 7 to 34% and key wood densities decreased between 14 to 25% (except at snow-dominated sites in the harvested dataset, which increased 43%; Table 6).

Random intercepts revealed that spatial correlation for repeated measures at the site scale and for sites within the same watershed was an important component in the variation structure. For example, a large proportion of variation in pool-spacing trends (47–52%) was explained by the random intercept for site in reference, harvested, and combined datasets, as was variation in large wood density in the combined and reference dataset (37–38%; Fig 6). However, more variation was explained by the watershed random intercept in key wood density trends in the combined (20%) and reference (27%) datasets relative to the harvested dataset (9%). The amount of variation explained by intrinsic landscape, hydrologic regime, and trend fixed effects was relatively low for all trend analyses (marginal $R^2 = 0.06–0.32$). The main effect of the year fixed effect explained the greatest proportion of variation for undercut banks, particularly in the combined and harvested analyses (marginal $pR^2 = 0.08–0.12$).

Fig 6. Proportion of variation explained by site and watershed random effects and fixed effects, in addition to the residual unexplained variation for best-supported trend models. The fixed effects portion includes any intrinsic landscape predictors, and if included, year and HydroClass. C: models for 4 response variables using the unfiltered dataset. R: models constructed using only data from reference sites with <6% subwatershed harvest and no

SAB harvest. H: models constructed using only data from harvested sites with >6% subwatershed harvest or >0% SAB harvest.

Discussion

Our exploration of a large, 30-year, forest-wide stream habitat dataset using type-3 analysis [24] revealed patterns in stream habitat metrics associated with spatial and temporal complexity in intrinsic landscape factors, forest management practices, and climate-driven hydrologic regimes. First, we found weak support for identifiable differences between previously investigated broad reference, harvested, and restored management classes that have been used to guide stream habitat management in the Tongass. The MRPP analysis revealed slightly stronger support for differences in channel process groups, such as floodplain compared with moderate-gradient channels. Therefore, we reanalyzed the data to explore nuanced relationships between intrinsic landscape factors and human actions associated with forest management and individual stream habitat metrics that are often targeted for improvement during restoration. Our results reaffirmed that catchment area (through discharge magnitude), and to a lesser effect, process group, are dominant factors in establishing a template for the formation of stream habitat features. Forest management-related predictors explained less variation, but several relationships had strong statistical support. Some major takeaways were intuitive, and others were surprising, including: 1. Sites with restoration post-harvest were significantly different from non-restored sites across a variety of metrics; 2. Pool-related features respond slowly to forest harvest and post-harvest restoration compared to more responsive habitat features such as wood densities; 3. Forest stand age is more important than total forest cover at watershed scales in explaining wood densities in

streams; 4. Spatial and temporal dimensions at which harvest has occurred (riparian vs. watershed, lag since harvest) are important considerations that were overlooked in previous management designs. Finally, temporal trends may foreshadow future trajectories including the concerning decline in vital channel-shaping key wood densities. Future experimental studies could inform our understanding of the mechanistic processes underlying these correlative patterns.

Assessment of broad management classifications as a means to characterize variability in stream habitat condition

The MRPP analysis and PCA ordinations showed greater separation in stream habitat characteristics based on natural landscape-scale variation described by process group than by broad categories of reference, harvested, or restored management classes. The dataset depicted a spatially heterogeneous forested landscape produced by a mosaic of past forest disturbances, where traditionally applied binary classifications of human management intensity (i.e., “reference” vs. “harvested” [7] may be becoming a less useful paradigm for guiding management decision-making. A more complete picture of landscape variability required the nuanced consideration of management types, ~~along with~~ intrinsic landscape-scale characteristics, and time. The analysis showed ~~how smaller, higher-gradient channels are predisposed to retaining key wood and forming a greater number of small pools, whereas larger floodplain streams have fewer, but larger,~~that smaller, higher-gradient channels are predisposed to retaining key wood and forming a greater number of small pools, whereas larger floodplain streams have fewer but larger pools. This complexity in landscape, management, restoration, and climate effects transcends what can be described by categorical process group and management history variables

alone and was a primary motivation for us to explore continuous variables describing new aspects of the Tongass NF landscape template (i.e., subwatershed area), specific types of management intensity (i.e., riparian or watershed harvest, and road construction), time for post-harvest forest regeneration, and spatial position of harvest relative to stream channels.

Role of the landscape template

In objective 2, our linear mixed model results inform our understanding of the landscape characteristics that influence stream habitats. Landscape characteristics also affect site accessibility and forest productivity for timber harvest and are likely reflected in the spatial structure of observed forest management predictors. Our models showed effects of subwatershed area and process group for most metrics, which integrate factors correlated with stream power (i.e., stream discharge, valley form, and slope) that have explained the greatest proportion of variation in stream habitat in previous studies in Washington and Oregon [26]. We found that D_{50} is not associated with management activities that deliver fine sediments to stream channels (Table 4), suggesting that sufficient stream power may exist to flush the bed surface of fine sediments [50]. Interestingly, the best-supported model for pool spacing still included subwatershed area despite the equation for pool spacing already adjusting for channel bed width (Table 3) [16]. This suggests that the~~re may be unexplained variation related to watershed size in the pool-spacing metric~~ pool-spacing metric may have unexplained variation related to watershed size.

Pool density and substrate transport may be heavily constrained by intrinsic landform characteristics on the Tongass NF. Similarly, other studies observed only weak relationships between management variables and pool densities [26], [27]. In contrast, Beechie ~~&~~ and Sibley

[51] found that greater wood densities could force a reduction in pool spacing and an increase in pool area that was dependent on channel slope. In our dataset, 55% of surveyed reaches may have an abundance of wood-forced pools given that they had pool spacing values <3 [16].

Management acting upon the template

After controlling for landscape factors, numerous relationships between stream habitat and forest management variables emerged. Effect sizes appeared generally small, but this may be attributable to changes in forest management practices over time and ~~that~~ the numerous regulatory mechanisms to protect stream habitats are working as intended on the Tongass. Strong declining trends exist in the harvest area at the subwatershed and RMA scales over the past 80 years, driven by market and regulatory factors (Fig 7). Owing to harvest regulation changes, harvest intensity has declined~~ with very few harvest events having occurred inside the RMA or exceeded~~, with very few harvest events occurring inside the RMA or exceeding 25% of a surveyed site's subwatershed area since 1990. This trend, along with changes in precipitation type and amount, ~~are one of several potential explanations driving trends in physical habitats that exist~~is one of several potential explanations driving trends in physical habitats in the data.

Fig 7. Harvest trends associated with surveyed streams in our dataset across the Tongass National Forest. "PP catchment" points indicate are the proportion of a study site's subwatershed that was harvested during an individual harvest event. "Buffer" points are the proportion of a site's SAB (91-m-wide buffer on each side of the stream) that was harvested in an individual riparian harvest event. Harvest events are defined as the amount

of harvest in a single year. Best-fit linear regression lines are displayed to illustrate declining harvest magnitudes over time at both spatial scales.

The largest effect sizes occurred for restoration status, highlighting it as a primary factor to consider when assessing stream habitat conditions. Some observed effects likely result from non-random selection procedures for restoration sites, rather than restoration outcomes *per se*. Stream restoration projects ~~nearly always were completed in large lowland reaches with historical riparian harvest that had documented~~were nearly always completed in large lowland reaches with historical riparian harvest documenting habitat deficiencies. This may suggest that managers were able to effectively identify and prioritize restoration actions at the sites that most needed it. Both large and key wood densities were similar in restored and unrestored sites because of wood additions during restoration that overcame key wood deficits. Without active restoration, ~~it may take hundreds of years for riparian forest~~riparian forests to regenerate and supply wood large enough to be recruited and retained in these wide, powerful lowland channels in SE Alaska [52], [53]. However, restored sites still had lower pool and undercut bank densities, and greater pool spacing, features that are important for overwintering juvenile salmonid survival [16]. This suggests that some habitat attributes like wood counts may be recovered instantly (coincident with the placement of wood during restoration activities), whereas others may change gradually over time [39], [54]. In our study, the average lag time from restoration to habitat survey was only 2.6 years. Sediment retention and scour processes may not have had adequate time to develop pools after wood installation, and documenting changes likely requires ongoing monitoring. Furthermore, some wood additions a~~re aimed at stabilizing existing pools rather than creating~~im to stabilise existing pools rather than create new ones.

Con formato: Fuente: Cursiva

Many studies have found decreased wood densities in stream reaches located in harvested watersheds compared to those with no harvest [52]. However, time since harvest is also important, with Murphy and Koski [55] reporting large wood densities in streams declining to a minimum 70 years following riparian timber harvest. In contrast, our models found no effect of SAB harvest on large wood densities at short lag times, but found higher large wood densities in reaches with large SAB harvest events 40–80 years before the survey date. It should be noted that because most riparian harvest occurred before 1990 and most habitat surveys occurred after 1990, there were only 78 surveys that had riparian harvest lags between 1 and 30 years. As a result, the dataset provides limited information to assess wood density response <40 years after a riparian harvest event. In headwater streams of SE Alaska, debris dams composed of small ~~wood pieces~~pieces of wood can form following riparian clear cuts that may initially increase wood densities [50]. However, this does not explain the lagged increase we observed, because these debris dams typically decompose rapidly and are flushed within 40 years [56]. More plausibly, mortality of red alder (*Alnus rubra*), a species common in disturbed riparian environments on the Tongass NF, grows quickly, reaching the threshold diameter for being considered LWD (>0.1 m) in <25 years. Alder mortality peaks at 90 years old, with the largest accumulations of hardwood woody debris occur~~ring 60–80 years post-harvest, thereby~~ 60–80 years post-harvest, aligning with the temporal relationships we observed [57].

Similarly, key wood densities were positively associated with the proportion of old-growth forest, suggesting that periodic recruitment of old-growth trees to stream channels is an important process in maintaining key wood densities. However, no relationships emerged for key wood with time since harvest. ~~It is likely that~~Likely, riparian regeneration 40–80 years following harvest had not yet produced trees with a large enough diameter to meet key wood thresholds

(these vary with stream size). These two findings ~~together~~ align with other studies that emphasize the importance of conserving old-growth forests as sources of key wood in streams because the regeneration of old-growth forest stands can take centuries. On the other hand, we did not find a decrease in key wood following riparian harvest. However, ~~there may be a long residence time of legacy coniferous key wood pieces~~legacy coniferous key wood pieces may have a long residence time that predate harvest [57]. Furthermore, the legally mandated 33-m RMB for fish-bearing streams on the Tongass NF did not provide total protection of the 91-m-wide SAB we used in analyses. Partial harvests of the SAB (post-1990) may have left narrow old-growth buffers vulnerable to windthrow~~ that provided~~, providing temporary supplemental sources of key wood recruitment for streams [39]. Nevertheless, riparian harvests could reduce key wood recruitment in the long term as mature riparian timber supplies dwindle so that streams have incurred a key wood debt in their riparian forests from past harvests that ~~has~~ have yet to be paid. Finally, the negative association between riparian harvest and undercut bank density aligns with previous studies that have also found fewer undercut banks in harvested habitats and suggest that riparian root mats can provide structural reinforcement [35], [58].

Comentado [A9]: check reference format

Our results illustrate how subwatershed harvest can interact with hydrologic processes, affecting sediment transport and some aspects of channel morphology. These results align with previous studies that showed stream channel widening relative to depth that may be caused by increased discharge peaks and bank destabilization following harvest [10]. After 1990, most harvest events on the Tongass NF were <30% of the subwatershed area. Harvest events ~~prior to 1990 encompassed on average 9.5% of the subwatershed area, declining to 3.8% post 1990, and there were~~before 1990 encompassed on average 9.5% of the subwatershed area, declining to 3.8% post 1990, and few harvests within the SAB recorded post-1990 (Fig 7). Smaller harvest

areas and increased protection of riparian buffers may explain the low effect sizes of harvest variables on channel-morphology metrics. For example, in a previous study, a 30% subwatershed harvest resulted in a modest annual increase in discharge of 14–20%, with changes persisting for 10–20 years [59]. These results may ~~provide support for~~support the effectiveness of more~~-~~ restrictive harvest regulations and practices, such as the Tongass Timber Reform act of 1990 in conserving stream habitat on the Tongass.

The importance of old-growth forest cover but not canopy cover at the subwatershed-scale in explaining large and key wood densities highlights that stand age and species composition may affect stream habitat. Even though some studies suggest 90% of the wood inputs to streams originate within <20 m of the stream channel [60], not all contributions are necessarily from local riparian stands. Episodic landslides can contribute to instream wood from upslope stands [61], and wood pieces may be transported as far as 2.5 km downstream in rivers of the Tongass NF [62]. Therefore, old-growth forests beyond the local riparian scale ~~have beneficial effects to stream-habitat complexity that~~hat benefit stream habitat complexity, which is important to fishes.

To our knowledge, this is the first study to examine wood densities based on snow- vs. rain-dominated hydrologies and therefore there is limited previous research to help explain why snow-dominated hydrologies may have more large wood and key wood compared with rain-dominated systems. Surprisingly, climate-related hydrologic regime was not associated with channel morphology or sediment transport-related metrics. It is possible that snow-dominated subwatersheds with greater capacity to store frozen precipitation may experience less extreme autumn and winter flooding and, therefore, less flushing of wood, allowing for wood to accumulate in these systems.

The results of our models demonstrated decreased pool quality, as measured by pool size and residual pool depth, in relation to road density within a subwatershed, aligning with studies that found lower residual pool depths in streams with road construction, timber harvest, livestock grazing, mining, and other forms of management [56]. Access road construction is often necessary for timber harvest to occur, and construction can facilitate overland runoff resulting in fine-sediment transport that can fill in pools. Although road crossings can be sources of sediment leading to aggradation in low-power channels or can be barriers to sediment transport if culverts are present, leading to downstream degradation (i.e., channel entrenchment or decreased width to depth ratios), we observed a weak negative association between width:depth and road-crossing density.

Habitat trends informing possible future condition

Pervasive effects on aquatic habitats resulting from underlying hydrologic processes that are currently changing may be important to consider when describing variation in habitat conditions on the Tongass NF [21]. Previous studies of environmental change in Alaska freshwater environments have mainly focused on dimensions of water quality and episodic disturbances, with a more limited focus devoted to trends in physical habitat that could change in lockstep with hydrology. The question of whether floods or drought fueled by climate change will act as temporary pulse disturbances for aquatic biota or whether they may inflict lasting press responses to stream ecosystems remains unanswered [63].

Temporal trends for undercut bank, large wood, and key wood densities may portend changes in habitat complexity that multiple life stages of resident and anadromous species depend on [64], [65]. Perhaps the strongest evidence for temporal trends in habitat metrics was

Comentado [A10]: Check

the contrasting trajectories of large and key wood, because both models are built with mostly the same survey data. Although the increase in large wood is somewhat encouraging, the decline in key wood over time is concerning. Complex low-velocity habitats are crucial for <u>the</u> life stages of some salmonids and decreasing key wood. Without active restoration through <u>the</u> installation of key wood in the channel, juvenile salmonid habitat may decline into the future.

In contrast, undercut bank density is an additional component of habitat complexity and the increasing trend we observed may partially offset losses in low-velocity refugia and cover from predation provided by key wood that supports salmonid populations [66]. The mechanism for increased undercut bank density requires additional investigation. Increased undercut banks in Coastal Range streams in Oregon have been linked to increased discharge in streams following watershed harvest with protected riparian buffers [67]. As previously discussed, larger runoff events on the Tongass may be related to climate change as increased winter rain-on-snow events may lead to bank scour, with riparian protections allowing well-developed root mats to stabilize undercutting banks.

The trends for habitat metrics we observed at reference sites, ~~which were~~ similar to those observed at harvested sites, cannot be attributed to changes in harvest practices because all reference sites had <6% historical timber harvest and no SAB harvest regardless of the date they were sampled. Comparison of habitat trends in rain- versus snow-dominated hydrologic regimes showed statistically detectable differences in trends for multiple metrics in combined reference and harvested datasets. The longitudinal sample size for this variable was more limited and explanations for such a pattern are unclear but could signal shifts in scouring intensity in lowland rain-dominated systems. Future monitoring could track the effects of such environmental change

Setting a course for the future management of northern coastal rainforests

Collectively, our results can help plan future research, habitat monitoring, and management for aquatic habitats in northern coastal forests. The ordination analysis highlighted how increased variation in harvest practices and timing has increased the heterogeneity of conditions within harvested stream sites, underscoring the value of our examination of continuous harvest variables and lag interactions in this analysis. However, increased detail in data collection could improve our understanding of these patterns and processes underlying stream habitat conditions. For example, rapid standardized assessments of riparian-zone conditions or the use of emerging technologies with high-resolution imagery or lidar could document tree species composition, stand age, and evidence of vulnerability to harvest-induced blowdown that could help managers assess future wood recruitment and retention potential [68]. Some rearing habitat occurs in floodplain ponds, but is not assessed in stream habitat surveys and may lie outside of buffered riparian areas. Inventory of other floodplain habitat features, such as beaver ponds, which have been shown to help sustain coho salmon recruitment [69], could also be incorporated into survey protocols. Large or key wood counts may be failing to capture information relevant to fish habitat. For example, Gomi et al.~~,~~ [56] found no significant difference in wood counts, but higher wood volume, in reaches flowing through old-growth forests compared to young forests. Measuring wood volume could be time consuming and prone to surveyor error, but surveyors may be able to classify large wood by species, wood type (i.e., conifer or deciduous), decay status, and stability to provide information on when wood recruitment occurred and to begin to flag streams at risk of future wood deficits.

Stream conditions may be changing independently from forest management in response to climate. Further, predicted changes in precipitation type could be severe for the region owing to the narrow thermal variability surrounding the freezing mark [70]. Flitcroft et al. [6] suggested that temporary decreases in young-of-the-year salmon populations in Tongass NF streams might have been related to extreme winter flooding that occurred in 2015. Synthetic stream models predict substrate scour [21] in response to shifts in hydrologic regimes and storm events. Such future conditions may make key wood an even more important habitat feature to maintain salmonid spawning gravels in high-power streams [21], [71]. Monitoring thereby may contribute to resilience of native salmonids.

Our work also has implications for habitat monitoring program design. The composite dataset that we used integrated data from multiple large-scale monitoring studies and is better suited for monitoring broadscale spatial patterns (e.g., [27]**)** rather than the mechanisms of habitat formation following disturbances (e.g., [56]), or temporal trends within specific repeatedly surveyed sites (e.g., [39]). Ongoing broadscale monitoring, however, does not necessarily require additional sites. Rather, simulations show that sampling larger numbers of sites in a landscape-scale study usually does not significantly improve the power to detect trends [72]. Spatially balanced and stratified rotating-panel survey designs could be used ~~to better~~better to monitor trends across reasonable spatial extents [73]. Further, the variance partitioning we conducted as part of our analyses (Fig 6) could be used in simulated power analyses (e.g., [72]) to design appropriate spatial extent (number of watersheds monitored), spatial resolution (number of sites within watersheds), stratification among different physiographic groups (geologies, topography, etc.) and temporal resolution (return rates) necessary to capture total variation across portions of the Tongass NF, although logistical constraints for accessing remote

sites may still hinder its implementation. For example, the greater site correlation for pool spacing suggests that there is less variance in this metric within sites over time, and longer return intervals may be appropriate to detect change in this variable.

Finally, our results highlighting the legacy of harvest on key wood that structurally supports pool formation and provides cover for juvenile salmonids may inform future harvest regulations. Forest management has trended toward protection using stream buffers with smaller individual harvest events (Fig 7). The 2012 Tongass National Forest Land and Resource Management Plan outlined plans to shift away from old-growth harvest, with a goal of at least 50% of total harvest being second-growth harvest by the year 2027 [33], and recently President Biden issued an executive order directing agencies to catalogue and end the harvest of old-growth forests on federal lands [74]. Widespread second-growth harvest is unprecedented on the Tongass NF, and consequences for floodplain and moderate-gradient streams are uncertain. In faster-growing low-latitude forests with ~~harvest rotations of 40–80 year~~40–80 years harvest rotations, reduced wood recruitment to streams has been documented, especially in large channels [52]. Short harvest return intervals may cut off long-term supplies of key wood even if narrow riparian buffers that are vulnerable to windthrow mortality are spared from harvest. The effects of second-generation harvest in high productivity, rain-dominated, marine-connected watersheds may disproportionately affect economically valuable species, including young coho salmon, which are more commonly associated with these streams, in contrast to resident cutthroat trout and sculpins (*Cottus* spp.), which are associated with interior snow-dominated systems [6]. Our results show the influence of watershed-scale old-growth forests, suggesting that management for aquatic habitat may benefit from the assessment of forest stand conditions at both riparian and subwatershed scales.

Acknowledgments:

Stream survey data was collected by hundreds of biologists, hydrologists and technicians employed by the Tongass National Forest and the PNW Research Station. Thanks to every one of you for your diligence in support of aquatic resources. Special thanks go out to our mentors – Steve Paustian, Dan Kelliher, Julianne Thompson, Rick Woodsmith, "Buck" Bryant and Brenda Wright who laid the foundations that we walk upon. Thank you to Kathryn Ronnenberg for assistance in copy-editing and improving and formatting figures. Any use of trade, firm, or product names is for descriptive purposes only and does not imply endorsement by the U.S. Government.

Data Availability Statement: At the time of publication, data were not publicly available from the U.S. Forest Service.

References

1. Rognerud S, Grimalt JO, Rosseland BO, Fernandez P, Hofer R, Lackner R, et al. Mercury and organochlorine contamination in brown trout (*Salmo trutta*) and arctic charr (*Salvelinus alpinus*) from high mountain lakes in Europe and the Svalbard archipelago. Water Air Soil Poll: Focus. 2002;2(2):209–232.
2. Edlund MB, Almendinger JE, Fang X, Hobbs JMR, VanderMeulen DD, Key RL, Engstrom DR. Effects of climate change on lake thermal structure and biotic response in northern wilderness lakes. Water. 2017;9(9):678.

3. Nie M. Governing the Tongass: national forest conflict and decision-making. Environ Law. 2006;36:385–480.
4. Berman M, Schmidt JI. Economic effects of climate change in Alaska. Weather Clim Soc. 2019;11(2):245–258.
5. Johnson AC, Bellmore JR, Haught S, Medel R. Quantifying the monetary value of Alaska national forests to commercial Pacific salmon fisheries. N Am J Fish Manage. 2019;39(6):1119–1131.
6. Flitcroft R, Munyon J, Claeson S, Johnson A, Moore M, Tucker E, Prussion K, Jacobson S, Lombard H. Forest legacies and climate realities: spatial and temporal variation in fish populations and habitat characteristics on the Tongass National Forest, Alaska. Gen. Tech Rep. PNW-GTR-1009. U.S. Department of Agriculture, Forest Service, Pacific Northwest Research Station, Portland, OR; 2022. 77 p.
7. Bryant MD, Caouette JP, Wright BE. Evaluating stream habitat survey data and statistical power using an example from southeast Alaska. North Am J Fish Manage. 2004;24(4):1353–1362.
8. Ross JA, Infante DM, Martin DJ, Rey M. Effects of riparian timber harvest on southeast Alaska stream habitat after 30–40 years: insights for management. N Am J Fish Manage. 2019;39(2):328–342.
9. Woodsmith RD, Buffington JM. Multivariate geomorphic analysis of forest streams: implications for assessment of land use impacts on channel condition. Earth Surf Proc Land. 1996;21(4):377–393.

10. Hetherington ED. Carnation Creek, Canada—review of a west coast fish/forestry watershed impact project. In: Proceedings of the Symposium of Forest Hydrology and Watershed Management, Vancouver, British Columbia; 1987. p. 531–538.
11. Murphy ML, Heifetz J, Johnson SW, Koski KV, Thedinga JF. Effects of clear-cut logging with and without buffer strips on juvenile salmonids in Alaskan streams. Can J Fish Aquat Sci. 1986;43:1521–1533.
12. Moore RD, Wondzell SM. Physical hydrology and the effects of forest harvesting in the Pacific Northwest: a review. J Am Water Resour As. 2005;41(4):763–784.
13. Dolloff CA. Effects of stream cleaning on juvenile coho salmon and Dolly Varden in southeast Alaska. Trans Am Fish Soc. 1986;115(5):743–755.
14. Martin DJ, Grotefendt RA. Stand mortality in buffer strips and the supply of woody debris to streams in southeast Alaska. Can J Forest Res. 2007;37(1):36–49.
15. Lisle TE. Sediment transport and resulting deposition in spawning gravels, north coastal California. Water Resour Res. 1989;25(6):1303–1319
16. Montgomery DR, Buffington JM, Smith RD, Schmidt KM, Pess G. Pool spacing in forest channels. Water Resour Res. 1995;31(4):1097–1105.
17. Schaberg PG, Hennon PE, D’Amore DV, Hawley GJ. Influence of simulated snow cover on the cold tolerance and freezing injury of yellow-cedar seedlings. Global Change Biol. 2008;14:1282–1293.
18. Sergeant CJ, Bellmore JR, McConnell C, Moore JW. High salmon density and low discharge create periodic hypoxia in coastal rivers. Ecosphere. 2017;8(6):e01846.

19. Bellmore JR, Fellman JB, Hood E, Dunkle MR, Edwards RT. A melting cryosphere constrains fish growth by synchronizing the seasonal phenology of river food webs. Global Change Biol. 2022;28:4807–4818.

20. Goode JR, Buffington JM, Tonina D, Isaak DJ, Thurow RF, Wenger S, Soulsby C. Potential effects of climate change on streambed scour and risks to salmonid survival in snow-dominated mountain basins. Hydrol Process. 2013;27(5):750–765.

21. Sloat MR, Reeves GH, Christiansen KR. Stream network geomorphology mediates predicted vulnerability of anadromous fish habitat to hydrologic change in southeast Alaska. Global Change Biology. 2018;23(2):604–620.

22. Leppi JC, Rinella DJ, Wilson RR, Loya WM. Linking climate change projections for an Alaskan watershed to future coho salmon production. Global Change Biology. 2014 Jun;20(6):1808–1820.

23. Bryant MD. Global climate change and potential effects on Pacific salmonids in freshwater ecosystems of southeast Alaska. Climatic Change. 2009 Jul;95(1–2):169–93.

24. Bisson PA, Gregory SV, Nickelson TE, Hall JD. The Alsea Watershed Study: A comparison with other multi-year investigations in the Pacific Northwest. In: Stednick JD, editor. Hydrological and biological responses to forest practices. Springer, New York, NY; 2008. p. 259–289. Springer, New York, NY.

25. Al-Chokhachy R, Roper BB, Archer EK. Evaluating the status and trends of physical stream habitat in headwater streams within the interior Columbia River and upper Missouri River basins using an index approach. Trans Am Fish Soc. 2010;139(4):1041–1059.

26. Anlauf KJ, Jensen DW, Burnett KM, Steel EA, Christiansen K, Firman JC, et al. Explaining spatial variability in stream habitats using both natural and management-influenced landscape predictors. Aquat Conserv. 2011b;21(7):704–714.

27. Steel EA, Muldoon A, Flitcroft RL, Firman JC, Anlauf-Dunn KJ, Burnett KM, Danehy RJ. Current landscapes and legacies of land-use past: understanding the distribution of juvenile coho salmon (*Oncorhynchus kisutch*) and their habitats along the Oregon Coast, USA. Can J Fish Aquat Sci. 2017;74(4):546–561.

28. Sergeant CJ, Falke JA, Bellmore RA, Bellmore JR, Crumley RL. A classification of streamflow patterns across the coastal Gulf of Alaska. Water Resour Res. 2020;56(2):e2019WR026127.

29. Deal RL, Orlikowska EH, D'Amore DV, Hennon PE. Red alder-conifer stands in Alaska: an example of mixed species management to enhance structural and biological complexity. Forests. 2017;8(4):131.

30. House of Representatives (H.R.) 987. (1990). Title II: Tongass National Forest Lands Protection. H.Rept 101-84 Part 2; S.Rept 101-261; H.Rept 101-84 Part 1; H.Rept 101-931.

31. Murphy ML. Forestry impacts on freshwater habitat of anadromous salmonids in the Pacific Northwest and Alaska: requirements for protection and restoration. (Decision Analysis Series No. 7). U.S. Department of Commerce, National Oceanic and Atmospheric Administration, Coastal Ocean Office, Silver Spring, MD; 1995.

32. Taylor RF. Yield of second-growth western hemlock-Sitka spruce stands in southeast Alaska. USDA Washington, D.C. Technical Bulletin No. 412; 1934.

33. USDA. Tongass Land and Resource Management Plan. R10-MB-769j; 2016.

34. Woodsmith RD, Noel JR, Dilger ML. An approach to effectiveness monitoring of floodplain channel aquatic habitat: channel condition assessment. Landscape and Urban Plan. 2005 Apr 30;72(1–3):177–204.

35. Bryant MD. A protocol using coho salmon to monitor Tongass National Forest land and resource management plan standards and guidelines for fish habitat. General Technical Report PNW-GTR-743. U.S. Department of Agriculture, Forest Service, Pacific Northwest Research Station, Portland, OR; 2008.

36. USDA. Alaska region aquatic habitat management handbook. Chapter 20. USDA Forest Service. FSH 2090.21. R-10 Amendment 2090.21-2001-1; 2001

37. Paustian SJ. A channel type users guide for the Tongass National Forest, Southeast Alaska. Technical Paper R10-TP-26, U.S. Department of Agriculture, Forest Service, Alaska Region, Juneau, AK; 1992.

38. McGarigal K, Wan HY, Zeller KA, Timm BC, Cushman SA. Multi-scale habitat selection modeling: a review and outlook. Landscape Ecology. 2016;31(6):1161–1175.

39. Martin DJ, Shelly A. Temporal trends in stream habitat on managed forestlands in coastal southeast Alaska. N Am J Fish Manage. 2017;37(4):882–902.

40. Oksanen J, Guillaume B, Friendly M, Kindt R, Legandre P, McGlinn D, et al. Vegan: community Ecology Package. R package version 2.5-7. https://CRAN.R-project.org/package=vegan; 2020.

41. McCune B, Grace JB. Analysis of ecological communities. MiM Software Design, Gleneden Beach, OR; 2002.

42. Dunn CG, Moore MJ, Sievert NA, Paukert CP, DiStefano RJ. Co-occurring lotic crayfishes exhibit variable long-term responses to extreme-flow events and temperature. Freshw Sci. 2021;40(4):626–643.

43. Bates D, Mächler M, Bolker B, Walker S. Fitting linear mixed-effects models using lme4. arXiv preprint arXiv:1406.5823. 2015.

44. Burnham KP, Anderson DR. Multimodel inference: understanding AIC and BIC in model selection. Sociol Methods Res. 2004;33(2):261–304.

45. Poff NL, Ward JV. Physical habitat template of lotic systems: recovery in the context of historical pattern of spatiotemporal heterogeneity. Environ Manage. 1990;14(5):629–645.

46. Gałecki A, Burzykowski T, Linear mixed-effects model. Springer New York; 2013.

47. Nakagawa S, Schielzeth H. A general and simple method for obtaining R2 from generalized linear mixed-effects models. Method Ecol Evol. 2013;4(2):133–142.

48. Stoffel MA, Nakagawa S, Schielzeth H. partR2: Partitioning R2 in generalized linear mixed models. bioRxiv. 2020. doi: 10.1101/2020.07.26.221168, https://www.biorxiv.org/content/10.1101/2020.07.26.221168v1.

49. Archer EK, Roper BB, Henderson RC, Bouwes N, Mellison SC, Kershner JL. Testing common stream sampling methods for broad-scale, long-term monitoring. General Technical Report RMRS-GTR-122. U.S. Department of Agriculture, Forest Service, Rocky Mountain Research Station, Fort Collins, CO. 2004.

50. Lisle TE. Stabilization of a gravel channel by large streamside obstructions and bedrock bends, Jacoby Creek, northwestern California. Geol Soc Am Bull. 1986;97(8):999–1011.

51. Beechie TJ, Sibley TH. Relationships between channel characteristics, woody debris, and fish habitat in northwestern Washington streams. Trans Am Fish Soc. 1997;126(2):217–229.
52. Beechie TJ, Pess G, Kennard P, Bilby RE, Bolton S. Modeling recovery rates and pathways for woody debris recruitment in northwestern Washington streams. North Am J Fish Manage. 2000;20(2):436–452.
53. Roper BB, Konnoff D, Heller D, Wieman K. Durability of Pacific Northwest instream structures following floods. N Am J Fish Manage. 1998;18(3):686–693.
54. Hartman GF, Scrivener JC, Miles MJ. Impacts of logging in Carnation Creek, a high-energy coastal stream in British Columbia, and their implication for restoring fish habitat. Can J Fish Aquat Sci. 1996;53(S1):237–251.
55. Murphy ML, Koski KV. Input and depletion of woody debris in Alaska streams and implications for streamside management. N Am J Fish Manage. 1989 Sep 1;9(4):427–436.
56. Gomi T, Sidle RC, Bryant MD, Woodsmith RD. The characteristics of woody debris and sediment distribution in headwater streams, southeastern Alaska. Can J For Res. 2001;31(8):1386–1399.
57. Andrus CW, Long BA, Froehlich HA. Woody debris and its contribution to pool formation in a coastal stream 50 years after logging. Can J Fish Aquat Sci. 1988;45(12):2080–2086.
58. Kershner JL, Roper BB, Bouwes N, Henderson R, Archer E. An analysis of stream habitat conditions in reference and managed watersheds on some federal lands within the Columbia River basin. N Am J Fish Manage. 2004;24(4):1363–1375.

59. Keppeler ET, Ziemer RR. Logging effects on streamflow: water yield and summer low flows at Caspar Creek in northwestern California. Water Resour Res. 1990;26(7):1669–1679.

60. McDade MH, Swanson FJ, McKee WA, Franklin JF, Sickle JV. Source distances for coarse woody debris entering small streams in western Oregon and Washington. Can J Forest Res. 1990;20:326–330.

61. Reeves GH, Burnett KM, McGarry EV. Sources of large wood in the main stem of a fourth-order watershed in coastal Oregon. Can J Forest Res. 2003;33(8):1363–1370.

62. Martin DJ, Benda LE. Patterns of instream wood recruitment and transport at the watershed scale. Transactions of the American Fisheries Society. 2001;130(5):940–958.

63. Lake PS. Disturbance, patchiness, and diversity in streams. J N Am Benthol Soc. 2000;19(4):573–592.

64. Bramblett RG, Bryant MD, Wright BE, White RG. Seasonal use of small tributary and main-stem habitats by juvenile steelhead, coho salmon, and Dolly Varden in a southeastern Alaska drainage basin. T Am Fish Soc. 2002 May 1;131(3):498–506.

65. Thorn WC. Validation of a trout habitat model for planning stream habitat improvement projects. Minnesota Department of Natural Resources, Fisheries Section; 1992.

66. Braun DC, Reynolds JD. Life history and environmental influences on population dynamics in sockeye salmon. Can J Fish Aquat Sci. 2014;71(8):1198–1208.

67. Bateman DS, Gresswell RE, Warren D, Hockman-Wert DP, Leer DW, Light JT, et al. Fish response to contemporary timber harvest practices in a second-growth forest from the central Coast Range of Oregon. For Ecol Manage. 2018;411:142–157.

68. Saarinen N, Vastaranta M, Honkavaara E, Wulder MA, White JC, Litkey P, et al. Mapping the risk of forest wind damage using airborne scanning Lidar. Int Arch Photogramm, Remote Sens Spatial Info Sci. 2015;40:189–196.
69. Pollock MM, Pess GR, Beechie TJ, Montgomery DR. The importance of beaver ponds to coho salmon production in the Stillaguamish River basin, Washington, USA. N Am J Fish Manage. 2004;24(3):749–760.
70. Shanley CS, Albert DM. Climate change sensitivity index for Pacific salmon habitat in southeast Alaska. PLoS ONE. 2014;9(8):e104799.
71. Buffington JM, Montgomery DR, Greenberg HM. Basin-scale availability of salmonid spawning gravel as influenced by channel type and hydraulic roughness in mountain catchments. Can J Fish Aquat Sci. 2004;61(11):2085–2096.
72. Larsen DP, Kaufmann PR, Kincaid TM, Urquhart NS. Detecting persistent change in the habitat of salmon-bearing streams in the Pacific Northwest. Can J Fish Aquat Sci. 2004;61(2):283–291.
73. Anlauf KJ, Gaeuman W, Jones KK. Detection of regional trends in salmonid habitat in coastal streams, Oregon. Trans Am Fish Soc. 2011a;140(1):52–66.
74. Exec. Order No. 14,072, 87 Fed. Reg. 129 (27 April 2022).

Supporting information captions

S1 Table. Minimum size thresholds for wood pieces to be considered “key wood”.

S2 Table. Descriptions of channel process group classes.

S1 Fig. Bar chart showing the number of best-supported univariate habitat models each predictor was included in out of 11 habitat variables that were modeled. Bar position and x-axis labels divide predictors into 4 groups. Spatial/temporal random effects, intrinsic landscape fixed effects, management fixed effects, and climate fixed effects.

S2 Fig. Beta coefficient estimates and their 95% confidence intervals for the two predominant process groups in the dataset (floodplain and moderate gradient) indicated by symbol type for seven habitat metrics where process group was retained in the best model.
