# Supplementary material for: Same streams in a different forest? Investigations of forest harvest legacies and future trajectories across 30 years of stream habitat monitoring on the Tongass National Forest, Alaska
Source: PLoS One. 2024 Jul 10;19(7):e0301723. doi: 10.1371/journal.pone.0301723 (PMC11236125; doi:10.1371/journal.pone.0301723)
Supplement: Attachment — Submitted filename: ADPResponsetoReviewPlosOne031624.docx [file pone.0301723.s006.docx]

**Response to Comments on Journal Requirements:**

When submitting your revision, we need you to address these additional requirements.

1. Please ensure that your manuscript meets PLOS ONE's style requirements, including those for file naming. The PLOS ONE style templates can be found at 
<https://journals.plos.org/>plosone/s/file?id=wjVg/PLOSOne_formatting_sample_main_body.pdf and 
<https://journals.plos.org/>plosone/s/file?id=ba62/PLOSOne_formatting_sample_title_authors_affiliations.pdf

We changed figure and supplemental figure format to match in-text names as described in the link above.

2. Please note that PLOS ONE has specific guidelines on code sharing for submissions in which author-generated code underpins the findings in the manuscript. In these cases, all author-generated code must be made available without restrictions upon publication of the work. Please review our guidelines at <https://journals.plos.org/>plosone/s/materials-and-software-sharing#loc-sharing-code and ensure that your code is shared in a way that follows best practice and facilitates reproducibility and reuse.

Code has been cleaned and prepared for release. It will be released through GitHub upon publication of the work.

3. Thank you for stating the following financial disclosure: "No external funding was used in the analysis represented by this manuscript, rather the United States Forest Service providing salary to support the involvement of all authors."

At this time, please address the following queries:

We were a bit confused by the requests for additional details on the topic of funding. See our best attempts to address them below.

a) Please clarify the sources of funding (financial or material support) for your study. List the grants or organizations that supported your study, including funding received from your institution.

The source of funding was the United States Forest Service. No external funding was used in this study.

b) State what role the funders took in the study. If the funders had no role in your study, please state: “The funders had no role in study design, data collection and analysis, decision to publish, or preparation of the manuscript.”

No external funders had any role in study design, data collection and analysis, decision to publish, or preparation of the manuscript.
c) If any authors received a salary from any of your funders, please state which authors and which funders.

All authors received salary through the US Forest Service.
d) If you did not receive any funding for this study, please state: “The authors received no specific funding for this work.”

NA

Please include your amended statements within your cover letter; we will change the online submission form on your behalf.

Included in cover letter.

4. In the online submission form, you indicated that "The data underlying the results presented in the study are available from Michael Moore mooremj@iastate.edu."

All PLOS journals now require all data underlying the findings described in their manuscript to be freely available to other researchers, either 1. In a public repository, 2. Within the manuscript itself, or 3. Uploaded as supplementary information.
This policy applies to all data except where public deposition would breach compliance with the protocol approved by your research ethics board. If your data cannot be made publicly available for ethical or legal reasons (e.g., public availability would compromise patient privacy), please explain your reasons on resubmission and your exemption request will be escalated for approval.

Michael Moore is now a USGS employee. To avoid conflicting data sharing rules in the USFS and USGS, we would like to establish Rebecca Flitcroft [rebecca.flitcroft@usda.gov](mailto:rebecca.flitcroft@usda.gov) as the primary contact for data and code for this research. The master dataset for this project is currently being reviewed for release through the US Forest Service’s repository, which will provide public access. Data will be available in repository upon publication.

5. We note that Figure 1 in your submission contain [map/satellite] images which may be copyrighted. All PLOS content is published under the Creative Commons Attribution License (CC BY 4.0), which means that the manuscript, images, and Supporting Information files will be freely available online, and any third party is permitted to access, download, copy, distribute, and use these materials in any way, even commercially, with proper attribution. For these reasons, we cannot publish previously copyrighted maps or satellite images created using proprietary data, such as Google software (Google Maps, Street View, and Earth). For more information, see our copyright guidelines: <http://journals.plos.org/>plosone/s/licenses-and-copyright.
 
We require you to either (1) present written permission from the copyright holder to publish these figures specifically under the CC BY 4.0 license, or (2) remove the figures from your submission:
 
     1. You may seek permission from the original copyright holder of Figure 1 to publish the content specifically under the CC BY 4.0 license.  
 
We recommend that you contact the original copyright holder with the Content Permission Form (<http://journals.plos.org/>plosone/s/file?id=7c09/content-permission-form.pdf) and the following text:
“I request permission for the open-access journal PLOS ONE to publish XXX under the Creative Commons Attribution License (CCAL) CC BY 4.0 (<http://creativecommons.org/licenses/by/4.0/>). Please be aware that this license allows unrestricted use and distribution, even commercially, by third parties. Please reply and provide explicit written permission to publish XXX under a CC BY license and complete the attached form.”
 
Please upload the completed Content Permission Form or other proof of granted permissions as an ""Other"" file with your submission.
 
In the figure caption of the copyrighted figure, please include the following text: “Reprinted from [ref] under a CC BY license, with permission from [name of publisher], original copyright [original copyright year].”
 
     2. If you are unable to obtain permission from the original copyright holder to publish these figures under the CC BY 4.0 license or if the copyright holder’s requirements are incompatible with the CC BY 4.0 license, please either i) remove the figure or ii) supply a replacement figure that complies with the CC BY 4.0 license. Please check copyright information on all replacement figures and update the figure caption with source information. If applicable, please specify in the figure caption text when a figure is similar but not identical to the original image and is therefore for illustrative purposes only.
The following resources for replacing copyrighted map figures may be helpful:
 
USGS National Map Viewer (public domain): <http://viewer.nationalmap.gov/viewer/>
The Gateway to Astronaut Photography of Earth (public domain): <http://eol.jsc.nasa.gov/sseop/clickmap/>
Maps at the CIA (public domain): <https://www.cia.gov/library/publications/the-world-factbook/index.html> and <https://www.cia.gov/library/publications/cia-maps-publications/index.html>
NASA Earth Observatory (public domain): <http://earthobservatory.nasa.gov/>
Landsat: <http://landsat.visibleearth.nasa.gov/>
USGS EROS (Earth Resources Observatory and Science (EROS) Center) (public domain): [http://eros.usgs.gov/#](http://eros.usgs.gov/)
Natural Earth (public domain): <http://www.naturalearthdata.com/>

6. Please upload a copy of Supporting Information Figure/Table/etc. S1 Table and S2 Table which you refer to in your text on page 54.

GIS Data used in figure 1 comes primarily from shapefiles we built from our own data from our analysis dataset that was collected by the USFS (now provided as supplementary info). The Alaska State Boundary Shapefile is owned by the United States Census and available for free use. The only other externally accessed GIS data would be the ArcGIS Pro World Ocean basemap. Basemaps can be used freely in academic publications as explained in ESRI online terms of use resources such as:https://doc.arcgis.com/en/arcgis-online/reference/static-maps.htm. and https://support.esri.com/en-us/knowledge-base/what-is-the-correct-way-to-cite-an-arcgis-online-basema-000012040, and through consultation with our USFS GIS specialist. In accordance with online guidelines for attribution and examples in other PlosOne publications, we added the following to the caption of Figure 1:

“Alaska state boundaries shapefile produced by the United States Census Bureau was accessed from: <https://www.census.gov/geographies/mapping-files/time-series/geo/carto-boundary-file.html>. ESRI World Oceans Basemap accessed from: https://www.arcgis.com/home/item.html?id=67ab7f7c535c4687b6518e6d2343e8a2;

Sources: Esri, GEBCO, NOAA, National Geographic, DeLorme, HERE, Geonames.org, and other contributors,” on lines 63-87 of the revised.

7. Please review your reference list to ensure that it is complete and correct. If you have cited papers that have been retracted, please include the rationale for doing so in the manuscript text, or remove these references and replace them with relevant current references. Any changes to the reference list should be mentioned in the rebuttal letter that accompanies your revised manuscript. If you need to cite a retracted article, indicate the article’s retracted status in the References list and also include a citation and full reference for the retraction notice.

We reviewed references and made a few minor changes to citation styles.

**Editor Specific Comments**

Title:
• Better the short title: “Impacts of Forest Harvest Legacies on Stream Habitat in the Tongass National Forest, Alaska”

Change made on lines 5-6 of the revised.

Materials and Methods:
• Include information about the restored and reference sites and provide details about the forests. Enhance the paper with relevant photos or illustrations that immerse the reader in the context of the forests. Additionally, incorporate more visual material related to the harvesting treatments, such as photos and illustrations.

We agree that a visual illustration of stream sites in different management categories would be beneficial. Therefore, we created a three paneled map with a representative example of each treatment. We included this as the new S1 fig for now and adjusted other SFig numbers, but are open to its inclusion in the main text if the editors think it would be better placed there. We cite it in text on line 289 of the revised and add a caption as well that reads: “**S1 Fig.** Examples of stream sites in the “reference”, “harvested”, and “restored” management categories. Panel A shows a stream in reference condition with no riparian buffer and limited subwatershed harvest. Panel B shows a site with substantial buffer and subwatershed harvest. Panel C shows a restored site that had buffer harvest and wood was installed to help improve stream habitat conditions. Collectively, these panels illustrate the multi-storied forest condition of reference stands, the conversion to Alder dominated riparian forest post-harvest that is discussed in the paper, and it shows some installed wood pieces at restoration sites.”

• Provide additional information about the stream`s characteristics and consider including a map displaying the streams or an illustrative example.

We left stream lines off of the figure 1 map because the stream density in the stream network is too dense to show any meaningful information. See comment and figure example in response to comment on line 175 below.

We add additional information describing the characteristics of the primary process groups and provide citations of Paustian’s classification framework and the Tongass Management plan where these are described in greater detail on lines 241-253 of the revised. The added text is:

“Floodplain systems are low-gradient alluvial depositional channels, situated in valley bottoms and lowlands with high stream flows not commonly contained within the active channel banks and some degree of flood plain development is evident. The channels are predominantly composed of series of pools and riffles, with large wood the predominant pool forming mechanism. They often have more multi-threaded channels, greater sinuosity, and greater amounts of off channel habitats such as beaver ponds and sloughs [33]. The moderate gradient channels usually have channel gradients of 2-6 percent and occur near the transition between headwater streams and floodplain and alluvial fan channels. These streams usually have more confined valleys and coarser alluvial substrates ranging from gravel to boulder size comprising the channel beds and banks. Large woody debris can form log-step pools and lateral scour pools in these channels [33]. Across process groups a variety of stream channel sizes are represented ranging from channel bed widths of 1.9 to 50.1 meters.”

We also provide representative photos in Figure S1.

• Expand on details about the floodplain.

See previous comment where floodplain and stream channels characteristics were described in greater detail.

• Enhance the information on the climate dataset, for example, specifying details like pixel size and the GIS geoprocessing involved.

The hydrologic classifications were developed by Sergeant et al. 2020. They classified stream flows from a complex runoff model for the Gulf of Alaska watershed that is based on a digital elevation model, landcover dataset, glacier inventory, and soil characteristics. We believe it is appropriate to add this short descriptor of the model and refer the reader to the source paper for a comprehensive presentation of the methodological details. The paper does not present the resolution of a raster model and summarizes the results to watersheds.

The revised sentence reads: “To capture differences in stream habitat that could be related to hydrologic regimes, we used published hydrologic classifications of rain-dominated and snow-dominated developed for watersheds in the Gulf of Alaska based on a classification of stream flows from a complex runoff model that incorporates a digital elevation model, landcover dataset, glacier inventory, and soil characteristics” On lines 278-285 of the revised.

• Relocate tables 1 and 3 to the appendixes

Made change as suggested and renumbered main-text and supplemental figures and tables

Statistical Analyses:
• Eliminate subtitles.

Deleted subtitles for statistical analyses.
• Clearly explain or provide more precise indications of the statistical analyses underlying Figures 4, 5, and 6. Consider moving relevant information from table and figure captions to the statistical analysis section.

We added text explaining analyses from the figure captions to statistical analysis sections as explained below. We do not believe that this information should be removed from these figure captions because figure captions should be able to stand alone with the reader being able to interpret them without consulting the methods section of the paper. Our USGS Bureau approving official requires this level of detail in figure captions.

Additional explanation of marginal effects calculations that are shown in figure 4: “We calculated marginal effect sizes with our top models in order to illustrate the magnitude of expected changes in habitat metrics in response to predictor variable changes. To do this we predicted the percent change in the habitat metric response variable to a 25% change of the observed range of a given continuous management or intrinsic predictor variable while holding all other continuous predictor variables constant at their mean. For binomial predictors such as restoration status or hydroclass, we predicted a percent change in response variables from one level to the other.” on lines 364-370 of the revised.

Note that Figure 4 illustrates the model results for a single response variable. We feel that it is more appropriate to explain the results and methods in the figure caption that directly corresponds to that particular variable than to explain a single model in the methods section.

For explanation of the plots in figure 5 representing the predicted marginal effect of year (i.e., temporal trends) we add the following to lines 390-392 of the revised. “We graphically present the marginal effect of survey year that may be indicative of temporal trends in the data by predicting the change in the response variable while holding all other continuous predictors constant at their mean.”

For explanation of fixed and random variation information in trend models in figure 6 we add: “We also report the conditional and marginal R^2^ for these trend models, which measures the proportion of variation explained by random- and fixed effects, respectively [47], using the partR2 package in R [48].” on lines 384-387 of the revised.

Results:
• Move Table 4 to the appendixes.
Made change as suggested

Discussion:
• Condense the “Management Acting Upon the Template” section to a concise summary (no more than four paragraphs) that succinctly conveys the key points and transitions smoothly to the main ideas.
• Include a section discussing the weaknesses of the study.
• Consider adding a conclusion section for a more comprehensive overview.

We recognize that the “Management Acting Upon the Template” section is long, but we believe the results are complex and there needs to be a brief discussion of all of the statistically and ecologically significant relationships we observed. This is essential in this study to provide this context and limitations of our findings, so that the findings are not taken out of context or misinterpreted by stakeholders. We streamlined the text in the revised version of this section but do not believe reducing it to 4 paragraphs is the most effective way to organize the distinct points being made. The section length was reduced from 1411 words to 1318 words.

As mentioned above, we believe we have already included discussion of the weaknesses and limitations of this study throughout the discussion rather than separating it into its own section. We believe it makes more sense to integrate the mention of our limitations in our ability to understand the effects of specific predictor variable alongside the discussion of the results of that variable. Some examples of limitations and weaknesses mentioned in the discussion follow.

“Some observed effects likely result from non-random selection procedures for restoration sites, rather than restoration outcomes *per se*. Stream restoration projects were nearly always completed in large lowland reaches with historical riparian harvest with documented habitat deficiencies” on lines 679-682 of the revised.

“In our study, the average lag time from restoration to habitat survey was only 2.6 years. Sediment retention and scour processes may not have had adequate time to develop pools after wood installation, and documenting changes likely requires ongoing monitoring. Furthermore, some wood additions aim to stabilize existing pools rather than create new ones, and thus would not change the number of pool at a given site.” On lines 691-696 of the revised.

“It should be noted that because most riparian harvest occurred before 1990 and most habitat surveys occurred after 1990, there were only 78 surveys that had riparian harvest lags between 1 and 30 years. As a result, the dataset provides limited information to assess wood density response <40 years after a riparian harvest event.” On lines 702-706 of the revised.

“Smaller harvest areas and increased protection of riparian buffers may explain the low effect sizes of harvest variables on channel-morphology metrics. For example, in a previous study, a 30% subwatershed harvest resulted in a modest annual increase in discharge of 14–20%, with changes persisting for 10–20 years [59].” On lines 739-744 of the revised.

Later in the “Setting a course for the future management of northern coastal rainforests” section we also discuss limitations and how future studies can address our shortcomings. Examples include:

“However, increased detail in data collection could improve our understanding of these patterns and processes underlying stream habitat condition. For example, rapid standardized assessments of riparian-zone condition or the use of emerging technologies with high-resolution imagery or lidar could document tree species composition, stand age, and evidence of vulnerability to harvest-induced blowdown that could help managers assess future wood recruitment and retention potential [68]. Some rearing habitat occurs in floodplain ponds, but is not assessed in stream habitat surveys and may lie outside of buffered riparian areas. Inventory of other floodplain habitat features, such as beaver ponds, which have been shown to help sustain coho salmon recruitment [69], could also be incorporated into survey protocols. Large or key wood counts may be failing to capture information relevant to fish habitat. For example, Gomi et al., [56] found no significant difference in wood counts, but higher wood volume, in reaches flowing through old-growth forests compared to young forests. Measuring wood volume could be time consuming and prone to surveyor error, but surveyors may be able to classify large wood by species, wood type (i.e., conifer or deciduous), decay status, and stability to provide information on when wood recruitment occurred and to begin to flag streams at risk of future wood deficits.” On lines 810-825 of the revised.

“The composite dataset that we used integrated data from multiple large-scale monitoring studies and is better suited for monitoring broadscale spatial patterns (e.g., [27]**)** rather than the mechanisms of habitat formation following disturbances (e.g., [56]), or temporal trends within specific repeatedly surveyed sites (e.g., [39]).” On lines 835-839 of the revised.

We add a brief conclusion section with main takeaways on lines 869-878 of the revised:

“Conclusion

Our analysis of a long-term dataset of stream geomorphology and habitat in a federally-managed temperate rainforest provides insights into broad-scale relationships between habitat condition, and past and present forest management practices, and climate-driven hydrology. Intrinsic and spatial variables explained most of the variation. The most important predictors of habitat metrics included stand age, harvest history, stream restoration among others. Surprising trends existed including increases in undercut banks and decreases in key wood densities that could be investigated further. Overall our results show the influence of watershed-scale old-growth forests, suggesting that management for aquatic habitat may benefit from the assessment of forest stand condition at both riparian and subwatershed scales.” On lines 863-872 of the revised.

**Reviewer 1 General Comments**

Reviewer #1: Dear author,
The manuscript entitled "Same streams in a diff­­erent forest? Investigations of forest harvest legacies and future trajectories across 30 years of stream habitat monitoring on the Tongass National Forest, Alaska" is a very interesting paper that captures the reader's attention. The paper is well written, and I think it is pleasant to read. This research emphasizes the imperative for sustainable practices to preserve fish habitats and maintain the overall health of southeast Alaska´s forest ecosystems. In my opinion, understanding these dynamics is crucial for anticipating and addressing future management challenges in this ecologically sensitive region. I enclose my specific comments in the PDF.
Bests

We thank the reviewer for their thoughtful and thorough review of our work!

**Reviewer 1 Specific Comments**

Line 34: add commas in “that some stream habitat metrics, such as pool densities, are…”

Change made on line 31 of the revised.

Lines 35 -37: Reword to “and management practices such as protecting riparian as well as post-harvest restoration may help”

Change made on line 32 of the revised.

Line 67: Can you provide a map of the streams?

At this scale chose not to provide a stream lines on the map. In the temperate rainforest climate of southeast Alaska there is a tremendous amount of precipitation that produces large amounts of runoff and very high stream densities in watersheds. The reader would not be able to distinguish the individual small streams well. See comment and figure example in response to comment on line 175.

Line 69: Check: the hydro class is a categorical variable

This is correct, hydroclass is categorical. Changed to “7 continuous variables and 1 categorical variable” on line 65 of the revised.

Line 110: Delete comma after “Bryant et al., [7]”

Made change on line 124 of the revised.

Line 121: Change “fluvial” to “stream”.

Accepted change on line 134 of the revised.

Line 123: Delete “as well as”

Accepted change on line 136 of the revised.

Line 125-126: If there were information about beaver densities or dam densities, it would be constructive for the analyses.

We do not think this information is available. We point out in the discussion that beaver pond inventories would be useful on lines 817-819 of the revised.

Line 140: insert “the”

Made change

Line 150: Check units “200 cm y^-1^”

This is correct. Rainfall can exceed 1110 cm, but across Tongass it is largely >200 cm/yr.

Line 173: Figure 1a does not show that information.

This sentence is talking about the total number of stream sites surveyed. The reference to Figure 1A refers to the map showing all of the sample sites. The reference to figure 1A isn’t about the remoteness of the sites. So, we moved the Figure 1A reference to earlier in the sentence to remove this confusion on line 185 of the revised.

Lines 175-176: Please add a map with relevant stream information for the manuscript’s goals, such as size, length, connectivity, and location.

There are a wide range of stream sizes and conditions. We tried our best to describe this in the site description portion of the manuscript. We were unclear how a map would add relevant information. We left stream lines off of the figure 1 map because the stream density in the stream network is too dense to show any meaningful information. See below for an illustration of what that would look like. Some surveyed streams are very small so it would be necessary to show all sizes. We added the supplementary figure S1 with visual illustration of some of our streams to hopefully provide some of this information.

Line 203: Lowercase “morphology”

Made change

Line 205: Change subhead to “Categorical land management classification”

Made the change on line 212 of the revised.

Line 209: Add comma after “therefore”

Made the change on line 216 of the revised.

Line 227: Insert “the”

Made the change on line 234 of the revised.

Line 242: Table 3 make “morphology” and “transport” lowercase.

Made the change and this table was moved to supplementary materials.

Line 250: Change “as well as” to “and”

Made the change on line 259 of the revised.

Line 262: insert “the” before “number”

Made the change on line 271 of the revised.

Line 286: add comma after “comparisons”.

Made the change on line 294 of the revised.

Lines 326-327: change “effects of spatial and temporal autocorrelation” to “spatial and temporal autocorrelation effects”

Made the change on line 332 of the revised.

Lines 376-377: Change “through the use of random spatial and fixed physiographic effects and by” to “ by using random and spatial and fixed physiographic effects and”

Made the change on line 389 of the revised.

Line 396: make “P-value” lowercase.

Made the change

Line 401: delete “more”

Made the change

Line 404: Add “a” in front of “substantial”

Made the change on line 414 of the revised.

Line 418: Figure 3. For PCA A, please include the sample and environmental variables that were considered. In other words, in addition to vectors, include the points (sites).

We decided to not include the site points in panel A so that variable vectors would be easier to see. All of the site points that the reviewer suggest we display are present in the other three panels. Therefore, we do not believe a change is warranted.

Line 429: Fig 1H. Check.

This is correct. Figure panel 1H scales site point size by forest cover as indicated in this line of text. No change is needed.

Line 453-454: make “Subwatershed” lowercase.

This actually needed a period to start a new sentence instead of a comma before “Subwatershed” so we added a period and retained the capitalization.

Line 476: Change “&” to “and”

Made the change

Line 479-481: Change “Channel morphology metrics and relative submergence were explained by fixed effects from multiple predictor groups,” to, “Fixed effects from multiple predictor groups explained channel morphology metrics and relative submergence.”

Made the change on line 493 of the revised.

Line 484: Change “densities of undercut banks and pools” to “undercut banks and pools densities”

We prefer to retain the original wording because the metrics describing undercut banks and pools are both per unit area or length so can be referred to as densities. In the suggested language “densities” seems to only apply to pools.

Lines 529-531: Change “Where a difference between rain and snow hydrologies was not supported for that dataset and response variable combination, plots are in grayscale” to “plots are in grayscale where a difference between rain and snow hydrologies was not supported for that dataset and response variable combination.”

Made change on lines 542-544 of the revised.

Line 620: Delete “along with”

Made change

Lines 621-623: Change “how smaller, higher-gradient channels are predisposed to retaining key wood and forming a greater number of small pools, whereas larger floodplain streams have fewer, but larger” to “that smaller, higher-gradient channels are predisposed to retaining key wood and forming a greater number of small pools, whereas larger floodplain streams have fewer but larger pools”

Made change on lines 633-635 of the revised.

Line 640: add period after (Table 4).

Made change on line 643 of the revised. Now it is S5 Table.

Lines 643-645: Change “there may be unexplained variation related to watershed size in the pool-spacing metric” to “pool-spacing metric may have unexplained variation related to watershed size.”

Made change on line 653 of the revised.

Line 648: change “&” to “and”

Made change on line 657 of the revised

Line 655: Delete “that”

Made change

Line 659-660: change “exceeded” to “exceeding”

Made change on lines 667 of the revised

Lines 662- 663: change “are one of several potential explanations driving trends in physical habitats that exist” to “is one of several potential explanations driving trends in physical habitats in the data.”

This sentence was ultimately deleted in trying to streamline this section as requested by reviewers.

Line 675: make “per se” italic.

Made change on line 680 of the revised

Line 676-677: change “nearly always were completed in large lowland reaches with historical riparian harvest that had documented” to “were nearly always completed in large lowland reaches with historical riparian harvest documenting”

We believe that the suggested wording is not appropriate. The stream restorations were not responsible for the documenting. The stream habitat restorations were completed where habitat deficiencies were documented. We changed to “were nearly always completed in large lowland reaches with historical riparian harvest with documented habitat deficiencies” on lines 681-682 of the revised.

Line 682: change “it may take hundreds of years for riparian forest” to “riparian forests”

We do not understand this suggestion as the suggested wording does not capture the intent of the sentence. The point is to emphasize the amount of time it takes for regeneration to occur. No change made.

Line 687: insert “the” after “coincident with”.

Made change on lines 690 of the revised

Line 691: change “are aimed at stabilizing existing pools rather than creating” to “aim to stabilize existing pools rather than create”

Made change on lines 695-696 of the revised

Line 703: change “wood pieces” to “pieces of wood”

Made change on line 707 of the revised

Line 709: change “Occurring 60-80 years post harvest, thereby” to “occur 60-80 years post-harvest”.

We believe “occurring” is correct as written. No change made.

Line 714: change “it is likely that” to “likely,”.

Changed to “possibly” to reflect that this is a hypothesis. Change on line 718 of the revised

Line 716: delete “together”

Made change on line 719 of the revised

Line 718: insert “the” after “because”

Made change on line 721 of the revised

Line 719: change “there may be a long residence time of legacy coniferous key wood pieces” to “legacy coniferous key wood pieces may have a long residence time”

Made change on line 723 of the revised

Line 724: change “that provided” to “providing”

Made change on line 727 of the revised

Line 727: change “has” to “have”

Made change on line 730 of the revised

Line 730: Check reference format.

We checked and believe this is correct.

Lines 735-737: change “prior to 1990 encompassed on average 9.5% of the subwatershed area, declining to 3.8% post 1990, and there were” to “before 1990 encompassed on average 9.5% of the subwatershed area, declining to 3.8% post 1990, and…”

Made change on lines 737-739 of the revised

Line 741: insert comma after “20%”

Made change on line 741 of the revised

Line 742: change “provide support for” to “support”

Made change on line 742 of the revised

Line 742: remove hyphen “more-restrictive”

Made change on lines 742- 743 of the revised

Line 743: Insert comma after “practices”

Made change on line 737 of the revised

Line 744: add period to end of sentence.

Made change on line 744 of the revised

Lines 751-752: replace “have beneficial effects to stream habitat complexity” with “benefit stream habitat complexity, which…”

Made change on lines 751-752 of the revised

Line 760: add commas after “and” and “therefore”

Made change on line 759 of the revised

Line 769: add comma after “present”

Made change on line 767 of the revised

Line 783: check citation format.

Checked and believe this to be correct

Line 786: add “the” before “life stages”

Made change on line 784 of the revised

Line 787: add “the” before “installation”

Made change on line 785 of the revised

Line 798: delete “which were”

Made change on line 796 of the revised

Line 810: Add “the” before heterogeneity

Made change on line 808 of the revised

Line 813: make “condition” plural

We think “condition” is appropriate as written. We are referring to the condition as in the habitat quality not general “habitat conditions”.

Line 814: make “condition” plural and add “the” after “or”

We think “condition” is appropriate as written. We are referring to the condition as in the habitat quality not general “habitat conditions”. However, we added “the” after “or”.

Line 822: delete comma after “al”\

Made change

Line 844: change “to better” to “better to”

We think that it is more appropriate as written. We are saying that these survey designs are not currently used but they could be used to better monitor trends. As suggested would imply that they are currently being used but could be improved.

Line 863: change “harvest rotations of 40- 80 years” to ’40-80 years harvest rotations”

Made change on line 861 of the revised but used “year” instead of “years”.

Line 868: add comma to after “species”

Made change on line 866 of the revised

Line 871: make “forest” plural

Made change on line 877 of the revised

Line 872: add “the” after “from” and make “condition” plural.

Added “the”. Again, we do not think “condition” should be plural as we are referring to condition as in quality.
